# Long-read sequencing reveals the complex structure of extra dic(21;21) chromosome and its biological effects

**DOI:** 10.1007/s00439-023-02583-9

**Published:** 2023-07-11

**Authors:** Kugui Yoshida-Tanaka, Ko Ikemoto, Ryoji Kuribayashi, Motoko Unoki, Takako Takano, Akihiro Fujimoto

**Affiliations:** 1 Department of Human Genetics, School of International Health, Graduate School of Medicine, The University of Tokyo, 7-3-1 Hongo, Bunkyo-Ku, Tokyo, 113-0033 Japan; 2 Department of Child Health, Tokyo Kasei University, 1-18-1 Kaga, Itabashi-Ku, Tokyo, 173-8602 Japan; 3 Tokyo Metropolitan Tobu Medical Center for Children with Developmental Disabilities, Tokyo, Japan

## Abstract

Complex congenital chromosome abnormalities are rare but often cause severe symptoms. However, the structures and biological impacts of such abnormalities have seldomly been analyzed at the molecular level. Previously, we reported a Japanese female patient with severe developmental defects. The patient had an extra dicentric chromosome 21 (chr21) consisting of two partial chr21 copies fused together within their long arms along with two centromeres and many copy number changes. In this study, we performed whole-genome, transcriptional, and DNA methylation analyses, coupled with novel bioinformatic approaches, to reveal the complex structure of the extra chromosome and its transcriptional and epigenetic changes. Long-read sequencing accurately identified the structures of junctions related to the copy number changes in extra chr21 and suggested the mechanism of the structural changes. Our transcriptome analysis showed the overexpression of genes in extra chr21. Additionally, an allele-specific DNA methylation analysis of the long-read sequencing data suggested that the centromeric region of extra chr21 was hypermethylated, a property associated with the inactivation of one centromere in the extra chromosome. Our comprehensive analysis provides insights into the molecular mechanism underlying the generation of the extra chromosome and its pathogenic roles.

**Supplementary Information:**

The online version contains supplementary material available at 10.1007/s00439-023-02583-9.

## Introduction

Constitutional chromosome abnormalities are a frequent cause of mental retardation and birth defects (Flint and Wilkie 1996). Aneuploidy and structural rearrangements of chromosomes account for most chromosome abnormalities, and many types of abnormalities, including monosomy, tetrasomy, chromosome translocations, and dicentric chromosomes, have been reported (McFadden and Friedman 1997; Morrow et al. 2018). However, the molecular mechanisms of phenotypic abnormalities and molecular changes caused by chromosome abnormalities have rarely been investigated (Morrow et al. 2018; Antonaros et al. 2021). We previously reported a partial tetrasomy 21 patient without typical Down syndrome (DS) phenotype (Takano et al. 2020). The female patient, who was 12 years old in 2023, was born to healthy Japanese parents (Takano et al. 2020), but her mother was 40 years old at the time of birth. The patient exhibited severe psychomotor retardation and multiple dysmorphic features but was not diagnosed with DS. Chromosome analyses revealed that the patient carried an extra chromosome 21 (chr21), which was dicentric and had two stalks on both ends; thus, her karyotype was 47,XX, + dic(21;21) (Takano et al. 2020). Extra chr21 consisted of two inverted duplications fused together within their long arms. An analysis of heterozygous single nucleotide polymorphisms (SNPs) using a DNA genotyping array showed that extra chr21 was of maternal origin. Whole genome sequencing (WGS) using short reads revealed that chr21 had regions with four and six copies, and the aneusomic region of chr21 had multiple structural variants (SVs). WGS also showed that extra chr21 lacked the DS critical region, which can explain the absence of the DS phenotype. Only five cases without the typical DS phenotype have been reported, including the present case.

Although our previous study investigated extra chr21 (Takano et al. 2020), further research is needed to fully understand its precise structure and molecular impact for several reasons. First, the previous study may have missed identifying SVs in repetitive genomic regions due to the limitations of short-read sequencing. Second, it did not provide the precise structure of complex SVs or the connection with copy number changes. Third, it did not analyze the impact extra chr21 has on gene expressions. Furthermore, since global aberrant DNA methylation has been observed in many cases of DS (Laufer et al. 2019), the DNA methylation status of extra chr21 deserves more attention.

Long-read sequencing may solve these problems. Long-read technologies have the potential to reveal accurate sequences of complex genomic regions and to identify a larger number of SVs than short-read technologies (Mantere et al. 2019). The longer read length also enables the direct reconstruction of haplotypes using heterozygous variants (Mantere et al. 2019). Furthermore, long reads can detect DNA methylation based on the pattern of electric current intensity (Liu et al. 2021). These technological improvements have revealed novel disease-causing mutations, the structures of complex SVs, and allele-specific DNA methylations, contributing to a better understanding of the mechanisms underlying diseases (Miller et al. 2021; Watson et al. 2022).

To understand the accurate structure of extra chr21 and the pathogenic changes it causes, we performed WGS, RNA sequencing (RNA-seq), and a DNA methylation analysis of the patient and her parents using long-read (Oxford Nanopore Technologies) and short-read sequencing (Illumina) technologies. Using our novel de novo localized assembly method (LoMA) (Ikemoto et al. 2023), we accurately determined the sequences of SVs and inferred the possible structures of extra chr21. Our allele-specific transcriptome analysis revealed that gene expression levels in the extra chr21 region were increased proportionally to the copy number. Additionally, an allele-specific DNA methylation analysis indicated that the methylation level of extra chr21 was higher than that of normal chr21. Taken together, our study provides critical insights into this complex chromosome abnormality and its biological impact.

## Materials and methods

### Clinical samples

The genomic DNA and RNA samples used in this study were obtained from a Japanese female patient with extra chr21 and her healthy parents with informed consent. The genomic DNA of the trio was extracted from peripheral blood leukocytes in our previous study (Takano et al. 2020). To obtain RNA samples, peripheral blood was collected from the patient and her mother at Tokyo Metropolitan Tobu Medical Center for Children with Developmental Disabilities. RNA was extracted from the peripheral blood using NucleoSpin RNA Blood (Takara Bio) according to the manufacturer’s protocol. RNA integrity numbers (RINs) were determined using an Agilent 4150 TapeStation (Agilent Technologies), and both samples had a high RIN (> 7).

Institutional Review Boards (IRBs) at the University of Tokyo and Tokyo Metropolitan Tobu Medical Center approved this work.

### WGS using long-read technology

WGS of the patient and her parents was performed using an Oxford Nanopore long-read sequencer. Libraries were prepared using an SQK-LSK110 Ligation Sequencing Kit (Oxford Nanopore Technologies) following the manufacturer’s protocol. Sequencing was carried out on FLO-MIN106 flow cells (Oxford Nanopore Technologies) using a MinION sequencer (Oxford Nanopore Technologies) for 96 h. Seven and one sequencing runs were performed for the patient and each parent, respectively. Reads with a mapping quality ≥ 60 were used for the following analyses. Copy numbers of the chr21 were estimated from the number of mapped reads within 500 bp bins.

### Identification of SVs

Base-calling was performed using Guppy V.4.4.1 (Oxford Nanopore Technologies) to obtain FASTQ files. The reads were mapped to the reference sequence (GRCh38) using Minimap2 (Li 2018), and BAM files were obtained from the mapping results. De novo SVs in the patient were detected using CAMPHOR somatic software (Fujimoto et al. 2021). In this analysis, clusters of reads with SVs were identified, and false positive candidates were removed by the mapping pattern (Fujimoto et al. 2021). SVs ≥ 100 bp and supported by ≥ 5 reads in the patient were detected. SVs detected within ± 300 bp of SVs in each parent or if the depth of coverage in each parent sample was ≤ 9 were excluded from the analysis. The breakpoints of the SVs were manually reviewed using Integrative Genomics Viewer (IGV).

### De novo assembly of the patient’s reads

To construct sequences of the regions with SV breakpoints accurately, we performed a de novo assembly using LoMA (Ikemoto et al. 2023). LoMA is software developed by us that produces highly accurate haplotype-resolved consensus sequences from erroneous long reads. We collected reads mapped within ± 20 kb of the SV breakpoints and conducted a de novo assembly using LoMA, which distinguished sequences of wild-type chromosomes and extra chr21 based on SVs. Consensus sequences generated by the software were aligned to the reference genome using BLAT (Kent 2002) to identify alterations in extra chr21.

### Classification of long reads

We previously reported that the patient’s extra chr21 is of maternal origin (Takano et al. 2020). To compare the expression and methylation patterns between extra and normal chr21, we classified reads from the patient as maternal and paternal using heterozygous SNVs (Fig. S1A). First, we selected “informative SNVs” (Fig. S1B) that were heterozygous in the patient and were (1) homozygous for different alleles (AA/BB) in the parents, (2) heterozygous for the father and homozygous for the mother (AB/BB), or (3) homozygous for the father and heterozygous for the mother (AA/AB) (Fig. S1B).

We analyzed the read sequences of each genomic position and classified the reads as either paternal or maternal based on the alleles of the informative SNVs (Fig. S1A). Reads were removed from further analysis if they contained multiple SNVs and the classification results were inconsistent among SNVs. Paternal and maternal reads were separately analyzed to compare gene expression and DNA methylation patterns between the extra (maternal) and normal chr21 (maternal and paternal).

### Analysis of variant allele frequency

The aneusomic region in extra chr21 contained regions with four and six copies (Takano et al. 2020); these regions affect the allele frequency of heterozygous SNVs. We analyzed the frequency of one allele of heterozygous SNVs in the patient’s short-read sequencing data, which we denoted as the B allele frequency (BAF) (Fig. S2).

### Fluorescence in situ hybridization* (FISH)*

Chromosome spreads were prepared from the patient's peripheral blood cells. Standard blood cultures were treated with ethidium bromide to obtain prometaphase cells. FISH was carried out using two SureFISH Chr21 CEP enumeration probes (P0, G0) and seven custom-designed probes (P1–P7) (Table S1, Agilent Technologies). Prometaphase spreads of the patient’s peripheral blood cells were hybridized with the probes, counterstained with 4',6-diamidino-2-phenylindole (DAPI), and analyzed using an epifluorescence microscope (modified protocol; https://www.agilent.com/cs/library/usermanuals/public/G9400-90000.pdf).

### Estimation of DNA methylation levels

We classified the reads based on their parental origin using SNVs (see above) and compared their DNA methylation status. We first classified the reads as maternal or paternal based on the informative SNVs. The methylation status of each cytosine at the CpG in each read was then estimated using Tombo software with the default setting (Oxford Nanopore Technologies) (Stoiber et al. 2016). We counted the number of methylated and unmethylated reads at each CpG site separately for maternal and paternal reads. For each CpG site, a 2 × 2 table was generated, consisting of the number of methylated maternal reads, the number of unmethylated maternal reads, the number of methylated paternal reads, and the number of unmethylated paternal reads. The proportion of methylated reads was compared between maternal and paternal reads using Fisher’s exact test (α = 0.001). CpG sites that exhibited a significantly larger number of methylated CpG sites in the maternal or paternal reads were defined as maternally or paternally hypermethylated sites, respectively. CpGs that covered less than 10 reads were excluded from this analysis.

We then compared the methylation status among various genomic regions, including simple repeats, long terminal repeats (LTRs), L1 transposons, *Alu* elements, alpha satellite repeats, genes, non-repeat intergenic sequences, promoters (1 kb upstream from the transcription start site of a gene), and centromeric regions (± 2 Mb of centromere) using repeat information obtained from the UCSC genome browser database (see URLs). We compared the proportion of maternally hypermethylated sites in these categories using the chi-square test (α = 0.05). Since chr21 is an acrocentric chromosome, we used the other acrocentric chromosomes (chr13, 14, 15, and 22) as controls.

## Results

### Copy number changes in the patient’s chr21 according to long-read sequencing

WGS of the patient using long reads yielded a total of 195 Gbp (65.0 × coverage) for the patient, 33.7 Gbp (11.2 × coverage) for the mother, and 34.4 Gbp (11.5 × coverage) for the father (Table S2). The depth of coverage showed a copy number oscillation in 21q, which was named the “oscillated region” (Fig. S3A). This result is consistent with our previous study (Takano et al. 2020). Based on the coverage of the disomic region (q22.11 → qter; mean: 58.44, SD: 13.41), we considered regions with depths between 31.61 and 85.26 (58.44 ± 13.41 × 2) as two-copy regions, between 90.05 and 143.69 (58.44 × 2 ± 13.42 × 2) as four-copy regions, and between 148.49 and 202.13 (58.44 × 3 ± 13.42 × 2) as six-copy regions.

**Fig. 1:**
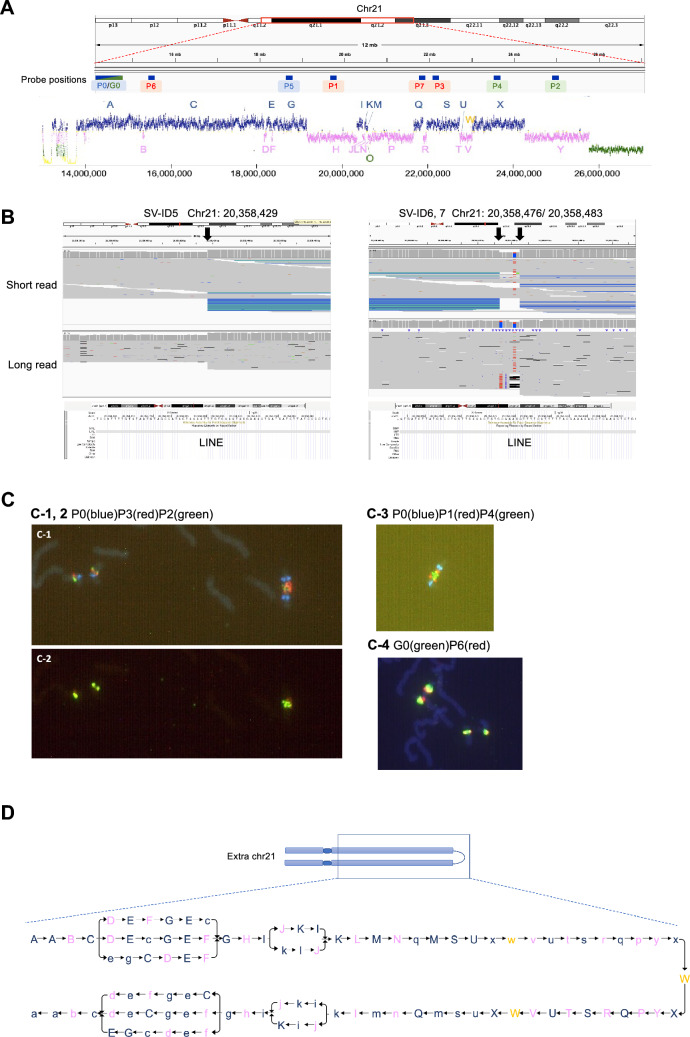
Structure of the extra chr21. **A** The mean depth of coverage in the oscillated region and the location of the FISH probes (P0–P7, G0) designed on chr21. Green, pink, and blue represent the depth of the coverage corresponding to two-copy regions (31.6–85.26), four-copy regions (90.05–143.69), and six-copy regions (148.49–202.13), respectively. Other values are indicated in yellow. The FISH probes were designed on blocks A, C, G, H, Q, S, X, and Y. **B** Visualization of short reads and long reads using IGV for the novel SVs. **C** FISH using P0, P1, P2, P3, P4, P6 and G0 probes. **C-1** Two normal chromosome 21 copies on the left and the dic(21;21) chromosome on the right are shown. Blue P0 signals near the centromere are located outmost, the green P2 signals are located inside, and red P3 signals are in between. **C-2** The P2 signals are slightly separated by a gap. **C-3** Blue P0 signals near the centromere are located outmost, followed by the red P1 signals. The green P4 signals are inside or close to the fused region, so there is no gap. The P1 signals are clearly visible because they are separated from P0 and P4. **C-4** Green G0 signals are near the centromere. A large black gap between the symmetric red P6 signals indicates that P6 is distant from the fused region. Yellow signals are the region where G0 and P6 appear to overlap. **D** A representation of the whole structure of the long arm of extra chr21 by connecting all possible patterns and the inverted version of each pattern on the other side of the symmetric structure

For a detailed analysis of the oscillated region, we calculated the average depth within 500 bp bins and defined the boundaries of the copy number changes (Fig. 1A, Table S3). Regions between boundaries were defined as copy number blocks and alphabetically labeled (Fig. S3B). While most of the blocks were four-copy or six-copy regions, block O was two-copy (1 kb) and block W was five-copy (3.5 kb) (Fig. S3C). The copy number in 21p could not be determined due to an insufficient number of mapped reads (mapping quality ≥ 60). However, based on the chromosome analysis showing that the patient’s extra chr21 had two centromeres and two stalks (Takano et al. 2020), we considered the 21p → cen region as tetrasomic (two copies in extra chr21 and two copies in normal chr21).

### Variant allele frequency within the patient

Short-read WGS identified 91,101 SNVs on chr21 in the patient, 91,924 in the mother, and 86,129 SNVs in the father. Among them, 16,405 were informative SNVs, and variant allele frequencies in the patient were analyzed from the short-read data (see “Classification of long reads” in Methods). Since extra chr21 was of maternal origin (Takano et al. 2020), the patient’s maternal alleles were expected to have higher frequencies in the oscillated region and to be 0.5 in the two copy (normal) regions. In this analysis, alleles of heterozygous SNVs in the patient are indicated by “A” and “B”, as shown in Fig. S1B, and the BAF was compared among regions (Fig. S2).

Among heterozygous SNVs in the mother, patterns of BAF were different between the six-copy and four-copy regions (Fig. S4, S5). The BAF distribution in the six-copy regions was trimodal, with peaks of 0.17 (1/6), 0.67 (4/6), and 0.83 (5/6), suggesting one, four, and five copies of B alleles (Fig. S4A, S4B). The BAF distribution in the four-copy regions was bimodal, with peaks of 0.25 (1/4) and 0.50 (2/4), suggesting one and two copies of the B allele, respectively (Fig. S4C, S4D). A higher BAF was observed in the 2.3 Mbp region indicated by the double-headed arrows in Fig. S4A and S4C. The pattern of allele frequencies indicates the heterogeneity of the maternal chromosomes.

### WGS using long reads

SVs were detected from the long-read WGS data. After excluding SVs found in the parents and a manual review (Fig. S6), we identified 15 patient-specific SVs on chr21 (Fig. 1B, Fig. S7, Table S4). Among these SVs, 10 were found in our previous study (Takano et al. 2020), while five were newly discovered through long-read sequencing (SV 6, 7, 9, 13, and 15) (Fig. 1B, Fig. S7). The five novel SVs were successfully validated by PCR (Fig. S8), and all 15 SVs were located within the oscillated region (Table S5). Although clear breakpoints were also observed for novel SVs in short reads, they were located within repeat regions or had complex structures, which would result in false negatives (Fig. 1B, Fig. S7). All breakpoints of the SVs were at the boundaries of the copy number blocks and were in intergenic or intronic regions.

To examine the breakpoint sequences of the SVs, we conducted a de novo local assembly of reads spanning each SV breakpoint using LoMA software (Ikemoto et al. 2023) (Supplementary information). Consensus sequences with SVs were successfully generated for 13 out of the 15 SVs and showed aberrant connections among the copy number blocks (Figs. S9, S10). To validate the accuracy of these consensus sequences, we performed Sanger sequencing of the PCR amplicons, and the obtained sequences perfectly matched the consensus sequence generated by LoMA (Fig. S8) (Supplementary information). These results suggest the reliability of our SV calling and the assembly approach using LoMA.

### Analysis of breakpoint sequences

The causes of SVs can be inferred from the structures of the breakpoints (Fujimoto et al. 2021; Kidd et al. 2010). To analyze these structures, we aligned the consensus sequences of the breakpoints to the reference genome sequences covering upstream and downstream regions (Fujimoto et al. 2021) and examined the homology length and insertion between breakpoints (Table S5, Fig. S9). SV-ID 2, SV-ID 3, and SV-ID 4 had no insertion between breakpoints but had microhomologies of 4 bp, 5 bp, and 5 bp, respectively (Fig. S9B–D). SV-ID 14 had microhomologies of 3 bp between the junction of blocks S and U, and 2 bp between the junction of blocks U and Y (Fig. S9K). All other SVs (SV-ID 5, 6, 7, 9, SV-ID 8, SV-ID 9, SV-ID 10, SV-ID 11, SV-ID 12, SV-ID 13, and SV-ID 15) had insertions at breakpoints (Fig. S9E–J, L).

Inserted sequences ≥ 20 bp were mapped to the reference genome with BLAT to determine their origins. Insertions in SV-ID 11 (93 bp) and SV-ID 13 (2710 bp) were aligned to multiple satellite repeat sequences (Fig. S9H, J). Four insertions from SV-ID 5, 6, 7, 9, SV-ID 8, and SV-ID 15 were aligned to the reference genome with high identity. Two of them (46 bp insertion of SV-ID 5, 6, 7, 9 and 42 bp insertion of SV-ID 8) were parts of long interspersed nuclear element (LINE) (Fig. S9E, F), and one (137 bp insertion of SV-ID 15) was a part of LTR (Fig. S9L). The other insertion (407 bp insertion of SV-ID 5, 6, 7, 9) was not a repeat and had a 6-bp microhomology with the subsequent sequence (Fig. S9E). Insertions in SV-ID 10 (49 bp), SV-ID 12 (26 bp), and SV-ID 15 (35 bp and 138 bp) were not found in the reference genome (Fig. S9G, I, L). SV-ID 12 had a duplication of 9 bp in block Z that was located on both sides of an insertion of 26 bp (Fig. S9I).

### Estimation of sequences of extra chr21

The determination of breakpoints for all junctions with copy number changes enabled us to estimate possible connections between the blocks estimated using the copy numbers and the SVs in extra chr21 (Fig. S11A). For example, three patterns of connections among blocks H, I, J, K, and L were possible (Fig. S11B, C). The consensus sequence of SV-ID 5, 6, 7, and 9 revealed the connection of blocks K (– strand) and I (+ strand) and of I (–) and K (+) (Fig. S9E, F). The consensus sequence of SV-ID 8 revealed the connection of blocks I (+) and K (–) and of K (+) and I (–) (Fig. S9E, F). Connections of the other blocks (blocks H and I, blocks I, J, and K, and blocks K and L) were considered the same as the reference genome. Therefore, after the successive connection of blocks H and I, connection to J (reference genome) or K (–) (SV-ID 8) is possible (Fig. S11C). In the former case, the order of the blocks can be as follows: J > K (order of reference genome) > I (–) (SV-ID 8) > K (SV-ID 5, 6, 7, 9) > L (order of reference genome) (Pattern 1). In the latter case, the order of the blocks can be: K > J (–) (order of reference genome) > I (–) > K (SV-ID 5, 6, 7, 9) > L (order of reference genome) (Pattern 2). Another possibility is the connection of I, J, K, and L as the reference genome (Pattern 3). Among these possible patterns, patterns 1 and 3 assumed one structural change (one insertion of I or K), whereas pattern 2 assumed three structural changes (two insertions and one inversion) (Fig. S11C). Therefore, patterns 1 and 3 were more plausible (Fig. S11C). This approach was also applied to the other regions (Fig. S11).

These possible patterns (Fig. S11B) indicate that block W, which is the only five-copy block, is one of the ends of the sequence (Figs. 1A, S3C). Additionally, the analysis of reads containing block X detected the connection of X (–) > W > X (Figs. S11D, S12). Considering the symmetric structure of extra chr21, block W would be the middle of the chromosome. Overall, the whole structure of the long arm of extra chr21 was estimated as two symmetric sets of rearranged four-copy regions and six-copy regions attached via the sequence of block W.

**Fig. 2:**
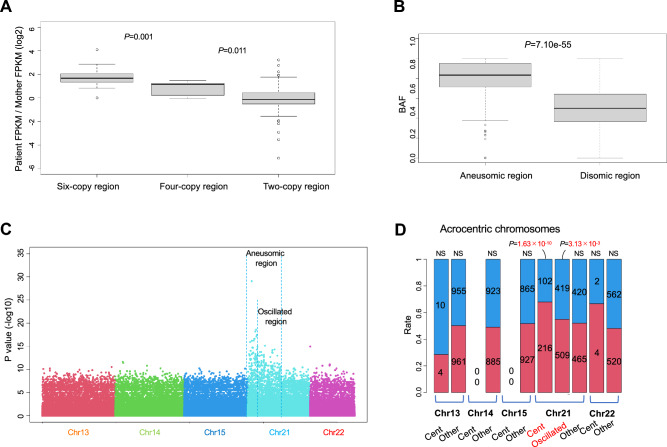
Gene expression and methylation patterns. **A** The ratio of gene expression levels in the oscillated region. Genes in the six-copy region showed a significantly higher ratio compared to genes in the four-copy region. Wilcoxon rank sum test. **B** B allele frequency (BAF) of heterozygous loci in expressed genes. The aneusomic region showed a significantly higher BAF compared to the disomic region. Wilcoxon rank sum test. **C** *P* values from Fisher’s exact test comparing the rates of methylated reads between the patient’s maternal and paternal reads among acrocentric chromosomes. A peak *P* value was observed in chr21. **D** Rates of maternally hypermethylated CpG sites (red) and paternally hypermethylated CpG sites (blue) that had significantly different methylation rates between maternal and paternal reads. The number in each bar shows the number of hypermethylated CpG sites. Chi-square test. *NS* not significant, *Cent* centromeric region, *Oscillated* oscillated region, *Other* other regions

We also performed FISH to validate the structure of extra chr21. Probes were designed as shown in Fig. 1A. Notably, FISH using the probe combinations of P0, P3, and P2 suggested that blocks A, S, and Y lie in this order (Fig. 1C). The signals of P2 (block Y) were slightly separated by a gap (Fig. 1C-2), while the signals of P4 (block X) were close without a gap (Fig. 1C-3), consistent with the y (–) > x (–) > W (+) > X (+) > Y (+) connection pattern (Fig. 1D). Other FISH results were consistent with our estimation; blocks A, Q, and X (Fig. S13A), blocks A, H, and X (Fig. 1C-3), blocks A and C (Fig. 1C-4), and blocks C, G, and X (Fig. S13B) lie in this order.

Based on the SVs and FISH, we inferred a plausible structure of extra chr21 (Fig. 1D).

### Overexpression of genes in the aneusomic region of chr21

To detect the changes in gene expression caused by extra chr21, we performed short-read RNA-seq for the RNA extracted from peripheral blood of the patient and her mother. RNA-seq yielded 15,472,000 reads and 18,048,000 reads, respectively (Supplementary Information). The ratio of gene expression levels (FPKM of the patient/FPKM of the mother) of the patient to her mother on chr21 indicated an increase in the expression of genes in the aneusomic region (median of the aneusomic region: 2.54, disomic region: 0.90; Wilcoxon rank sum test, *P* = 1.28 × 10^–10^) (Fig. S14A). Thirty-five genes were expressed in the oscillated region, including 11 protein-coding genes and 11 non-coding RNA genes (Fig. S14B). Of the 35 genes, 25 were in the six-copy regions, and 10 were in the four-copy regions (Table S6). The ratio of the gene expression level was significantly higher in the six-copy regions than in the four-copy regions and in the four-copy regions than in the two-copy regions (median of six-copy regions: 3.19, four-copy regions: 2.22, two-copy regions: 0.90; Wilcoxon rank sum test, *P* = 0.001, *P* = 0.011) (Fig. 2A). Additionally, we conducted quantitative PCR (qPCR) for seven genes (*RBM11, CXADR, BTG3 C21orf91, NRIP1, ATP5PF* and *GABPA*) that are functionally associated with the patient's phenotypes (see discussion). All seven genes exhibited higher expression levels in the patient compared to her mother (Fig. S14C). These results indicate that the gene expression level in the region of extra chr21 was elevated in comparison to normal regions.

We further sequenced the RNA of the patient and her mother using long-read sequencing (Supplementary Information, Table S7). Long reads identified three patient-specific transcripts (Kiyose et al. 2022) (Table S8). Two of them were known transcripts of *RNA binding motif protein 11* (*RBM11*:ENST00000400577.4 and ENST00000468643.5), a protein-coding gene located in the oscillated region. The third transcript was a novel transcript of *ATP binding cassette subfamily C member 13* (*ABCC13*), a pseudogene also located in the oscillated region.

### High maternal allele frequency of overexpressed genes

Since extra chr21 was of maternal origin (Takano et al. 2020), we compared the expression levels of genes from the paternal and maternal chromosomes. We analyzed the RNA-seq reads and compared the BAF of 705 informative SNVs in the expressed genes in the patient’s chr21 (Fig. S14D). Of these, the BAF of 321 loci were significantly different from 0.5 (*P* < 0.05), and 253 loci (78.8%) had a BAF significantly higher than 0.5. In the oscillated region, there were 201 informative SNVs. Of these, the BAF of 144 loci were significantly different from 0.5 (*P* < 0.05), and 138 loci (95.8%) had a BAF significantly higher than 0.5 (Fig. S14E). The lowest *P* value was observed at an SNV (chr21:14,967,249) in *nuclear receptor interacting protein 1* (*NRIP1*), in which 100 maternal reads and 13 paternal reads were observed (*P* = 2.74 × 10^–16^). The BAF of the aneusomic region (median; 0.83) was higher than in the disomic region (median; 0.50) (*P* = 7.10 × 10^–55^) (Fig. 2B). These results indicate that the genes on the maternal haplotype had higher expression levels than genes on the paternal haplotype.

### Maternal hypermethylation of maternal chr21

We classified long reads as paternal or maternal and compared the methylation rate of CpG sites (Fig. S15A). In chr21, particularly within the oscillated region, many CpG sites exhibited low *P* values compared to those in other acrocentric chromosomes (chr13, 14, 15, and 22) (Fig. 2C). Then we compared paternally hypermethylated CpG sites and maternally hypermethylated CpG sites across different regions, finding a significantly higher number of maternally hypermethylated CpG sites in the centromeric and oscillated regions (*P* = 1.63 × 10^–10^ and *P* = 3.13 × 10^–3^, respectively) (Table S9, Fig. 2D). No significant differences were observed in other chromosomes.

We further compared the number of paternally and maternally hypermethylated CpG sites among different genomic features in the centromeric and oscillated regions. In the centromeric region, significantly larger numbers of maternally hypermethylated CpG sites were observed in simple repeats, *Alu* elements, and centromeric alpha satellite repeats (*P* = 6.04 × 10^–4^, *P* = 4.02 × 10^–5^, and *P* = 9.11 × 10^–4^, respectively) (Fig. S15B, Table S10). On the other hand, in the oscillated region, significantly larger numbers of maternally hypermethylated CpG sites were observed in L1 transposons (90 maternal sites vs. 58 paternal sites), genes (196 maternal sites vs. 145 paternal sites), and non-repeat intergenic regions (490 maternal sites vs. 406 paternal sites) (*P* = 0.01, *P* = 0.01, and *P* = 0.01, respectively) (Table S11, Fig. S15C).

## Discussion

In the present study, we conducted genome, transcriptome, and DNA methylation analyses of a patient with an extra chr21 using both short-read and long-read sequencing data (Takano et al. 2020). To our knowledge, few studies have performed a comprehensive analysis of complex chromosome abnormalities using long-read sequencing technology.

The pattern of BAF obtained from heterozygous SNVs was consistent with the copy number changes, indicating the reliability of this analysis (Fig. S4). Our analysis of BAF provided insights into the origin of extra chr21. We observed a high BAF in a 2.3 Mbp region away from the pericentromeric region (double-headed arrows in Fig. S4A, S4C). This BAF pattern (Pattern 3 in Fig. S4B) indicates that the pericentromeric regions in both normal chr21 and extra chr21 have the same haplotype. However, we observed a change in BAF around chr21:16,372,000 (Fig. S4A), which is consistent with Pattern 2 (Fig. S4B). This change suggests a different haplotype between the pericentromeric and other regions, which implies a recombination event on extra chr21 near chr21:16,372,000.

Long-read sequencing detected SVs at all boundaries of the copy number changes and de novo assembly reconstructed sequences of the breakpoints. It is known that various mechanisms can lead to SVs, such as non-homologous end joining (NHEJ), alternative end joining (alt-EJ), non-allelic homologous recombination (NAHR), and fork stalling, template switching, and microhomology-mediated break-induced repair (FoSTeS/MMBIR) (Fujimoto et al. 2021; Kidd et al. 2010). Estimating the origin of SVs is possible based on the length of the homology and insertions at breakpoints. Among the identified SVs, seven had insertions > 10 bp (SV-ID 5, 6, 7, 9 were considered one event), five had 2–100 bp homology in the breakpoints, and one had no insertion or homology (Fig. S9, Table S5). Based on these results, we concluded that seven SVs were caused by FoSTeS/MMBIR, four by alt-EJ, and one by NHEJ (Fig. S9). A previous study suggests that NAHR, FoSTeS, MMBIR and telomeric fusions can generate dicentric chromosomes (Barra and Fachinetti 2018). However, NAHR and telomeric fusion were not observed in extra chr21. These findings suggest that multiple mechanisms generated the SVs in extra chr21.

Fig. S16 shows a model of the extra chr21 formation suggested by the present study. The analysis of BAF indicated that the three centromeres of the patient’s maternal chromosomes originated from the same haplotype (Fig. S4A, S4B). Since non-disjunction in meiosis is unlikely to result in three derivatives from a single chromosome, we propose that the non-disjunction of chr21 in an oocyte of the mother was the initial event. Then, the accumulation of SVs in a chr21, possibly caused by a breakage-fusion-bridge cycle (Takano et al. 2020), followed by replication and fusion of the abnormal chr21, occurred, resulting in extra chr21 with a symmetric structure.

We conducted a transcriptome analysis to find the quantitative and qualitative changes in transcripts using both short and long reads. By analyzing the short reads, we found a copy number-dependent upregulation of the genes on chr21 in the patient (Fig. 2A). While this upregulation and the gene-dosage compensation of gene expressions have been reported in DS (Aït Yahya-Graison et al. 2007; Antonaros et al. 2021; Mao et al. 2003), no compensation was observed in this case. We identified 11 protein-coding genes that showed an increased expression. Among them, four genes, *RBM11*, *CXADR Ig-Like Cell Adhesion Molecule* (*CXADR*), *BTG anti-proliferation factor 3* (*BTG3*), and *chromosome 21 open reading frame 91* (*C21orf91*), are related to neurogenesis (Li et al. 2016, 2018; Liu et al. 2022; Pedrotti et al. 2012; Rost et al. 2004; Slavotinek et al. 2000; Yoshida et al. 1998). Three other genes, *NRIP1*, *ATP synthetase peripheral stalk subunit F6* (*ATP5PF*), and *GA binding protein transcription factor subunit alpha* (*GABPA*), are associated with hypotonia in DS, which could be related to the patient’s severe delay in motor development (Brault et al. 2015). Our long-read RNA-seq data identified a patient-specific transcript in the *RBM11* gene (Table S8). RBM11 is a tissue-specific splicing factor during neuron and germ cell differentiation (Pedrotti et al. 2012). The expression change of *RBM11* may have affected the neural development of the patient, resulting in pathological phenotypes.

The chromosome analysis in our previous study revealed that the patient’s extra chr21 is dicentric (Takano et al. 2020). In general, a chromosome with two centromeres cannot be stably transmitted to daughter cells because such a chromosome is pulled to opposite poles during mitosis. Therefore, inactivation of one of the two centromeres by deletion or epigenetic modification is required for stable segregation, as seen in Robertsonian translocations (Earnshaw et al. 1989; Sullivan and Schwartz 1995). Since the patient’s extra chr21 is stably transmitted to daughter cells (Takano et al. 2020), we hypothesized that one of the two centromeres is epigenetically inactivated. Our allele-specific methylation analysis showed that the pericentromeric regions of the maternal chr21 of the patient were hypermethylated compared to that of the paternal chr21 (Fig. 2C, D). This observation supports our hypothesis. Additionally, this analysis showed hypermethylation in the oscillated region of the maternal chr21 (Fig. 2C, D). It is well known that one of the two X chromosomes is inactivated by a dosage compensation mechanism that includes DNA methylation in female cells. Several reports have shown that the dosage compensation response could occur for any type of aneuploidy (Disteche 2016). Hence, it is possible that such a mechanism caused the hypermethylation in the oscillated region.

This is the first comprehensive study of a complex chromosome abnormality using long-read sequencing and novel bioinformatics methods. Thus, it successfully revealed the structures of all junctions associated with copy number changes, reconstructed the sequence of extra chr21, and examined the methylation changes occurring in extra chr21. Additionally, it demonstrated alterations in gene expressions and methylations caused by the presence of extra chr21. These findings provide an accurate characterization of the structure of extra chr21, shed light on the associated epigenetic changes, and suggest potential mechanisms underlying the generation of this abnormal chromosome.

## URLs

Picard: http://broadinstitute.github.io/picard/. UCSC: https://hgdownload.soe.ucsc.edu/goldenPath/hg38/database/. refSeq gene: https://hgdownload.cse.ucsc.edu/goldenpath/hg38/database/refGene.txt.gz. RepeatMasker: https://hgdownload.cse.ucsc.edu/goldenpath/hg38/database/rmsk.txt.gz. Simple repeat: https://hgdownload.cse.ucsc.edu/goldenpath/hg38/database/simpleRepeat.txt.gz. Centromere: https://hgdownload.cse.ucsc.edu/goldenpath/hg38/database/centromeres.txt.gz. Tombo: https://github.com/nanoporetech/tombo

## Supplementary Information

Below is the link to the electronic supplementary material.

Supplementary file1 (PDF 27501 KB)

Supplementary file2 (PDF 379 KB)

## Data Availability

The datasets generated and analyzed during the current study are available from the corresponding author on reasonable request.
