# Supplementary material for: Long-read sequencing reveals the complex structure of extra dic(21;21) chromosome and its biological effects
Source: Hum Genet. 2023 Jul 11;142(9):1375–84. doi: 10.1007/s00439-023-02583-9 (PMC10449678; doi:10.1007/s00439-023-02583-9)
Supplement: Supplementary file 1 — Supplementary file1 (PDF 27501 KB) [file 439_2023_2583_MOESM1_ESM.pdf]

# Supplementary Information

Long-read sequencing reveals the complex structure of extra dic(21;21) chromosome and its biological effects

Kugui Yoshida-Tanaka[1], Ko Ikemoto[1], Ryoji Kuribayashi[1], Motoko Unoki[1], Takako Takano[2,3], and Akihiro Fujimoto[1]

1 Department of Human Genetics, School of International Health, Graduate School of Medicine, The University of Tokyo, Tokyo, Japan

2 Department of Child Health, Tokyo Kasei University, Tokyo, Japan

3 Tokyo Metropolitan Tobu Medical Center for Children with Developmental Disabilities, Tokyo, Japan

Corresponding author:

Takako Takano

Department of Child Health, Tokyo Kasei University

1-18-1 Kaga, Itabashi-ku, Tokyo 173-8602, JAPAN

TEL & FAX: +81-3-3961-5339

E-mail: takano@tokyo-kasei.ac.jp

Akihiro Fujimoto

Department of Human Genetics, The University of Tokyo, Graduate School of Medicine,

7-3-1 Hongo, Bunkyo-ku, Tokyo, 113-0033, JAPAN

TEL: +81-3-5841-3692

E-mail: afujimoto@m.u-tokyo.ac.jp

**Supplementary Methods**

*WGS using short-read technology*

WGS data of the patient acquired in our previous study[1] was used in this study. One microgram of genomic DNA of the parents was subjected to WGS. Libraries were prepared using a TruSeq Nano DNA Library Kit (Illumina) and sequenced using a NovaSeq6000 (Illumina).

*Validation of SVs*

Our analysis identified SVs that were not detected by short-read sequencing in our previous study[1]. To validate these novel SVs, PCR primers were designed based on consensus sequences reconstructed by LoMA[2] (SV-ID 6, 7, and 9 and SV-ID 13) or the sequence of a read with an SV (SV-ID 15). The regions spanning SV junctions were amplified by PCR using the KOD Multi&Epi enzyme (TOYOBO), and the amplicons were subjected to Sanger sequencing.

*Variant call for short-read WGS*

Read sequences were mapped using Burrows-Wheeler Aligner (BWA)[3] to the human reference genome (GRCh38). Possible duplicate reads generated by PCR were removed using Picard MarkDuplicates. SNVs and short insertions and deletions (indels) were detected using GATK HaplotypeCaller[4].

*RNA-seq using short reads and estimation of gene expression levels*

One microgram of total RNA from the patient and her mother was used for the library preparation and short-read sequencing. Libraries were prepared using a TruSeq stranded Total RNA/Ribo-zero Kit (Illumina) and sequenced on the NovaSeq6000.

The reads were mapped to GRCh38 using HISAT2 (v2.1.0)[5], and the read counts were obtained using featureCounts (v2.0.2)[6]. The read counts from the two samples were converted to fragments per kilobase of exon per million reads mapped (FPKM), and quantile normalization was performed using the preprocessCore package in R (v4.0.3). To obtain reliable gene expression results, we removed genes with very low expression levels (FPKM less than 0.31) based on a previous study[7]. To compare the expression levels, we calculated the ratio the patient FPKM to mother FPKM for each gene.

We then compared the expression patterns of transcripts from the maternal and paternal chromosomes of the patient. We selected RNA-seq reads with a mapping quality $\geq 60$ and $10 \leq$ coverage $< 1000$. The reads were classified as either paternal or maternal in origin based on heterozygous SNVs (Fig. S1). The numbers of maternal and paternal

reads were compared using a chi-square test for each SNV (α=0.05). We further compared the BAF between the aneusomic region (chr21:1-25,790,500, region containing extra chr21) and disomic region (chr21:25,790,501–46,800,000, region containing normal chr21) of chr21 using the Wilcoxon rank sum test (α=0.05).

*Examination of gene expression levels using qPCR*

Total RNA was reverse-transcribed using a PrimeScript RT Reagent Kit with gDNA Eraser (Takara). The resulting cDNA was used for qPCR with a KAPA SYBR Fast qPCR kit (NIPPON Genetics) according to the manufacturer's instructions. *ACTB* expression was used as a reference gene for normalization. Each sample was analyzed in triplicate, and the average relative quantification (RQ) values (target gene/ACTB) were calculated. Statistical significance of the RQ values between the patient and the mother was assessed using the one-sample t-test. The PCR primer sequences used in this study are listed in Table S12.

*RNA-seq using long reads and the annotation of transcripts*

One microgram of total RNA was extracted from the patient and her mother, and cDNA was synthesized using the SMARTer PCR cDNA Synthesis Kit (Clontech). Libraries were prepared using the SQK-LSK110 Ligation Sequencing Kit following the manufacturer's protocol. The libraries were sequenced on FLO-MIN106 flow cells using a MinION sequencer for 96 hours.

Base-calling of the two samples was performed using Guppy V.4.4.1. Read sequences were analyzed using SPLICE software[8] to identify splicing variants.

## Supplementary Results

*Detection of SVs*

SVs were detected from the long-read WGS data. After removing the SVs found in the parents, 19 SVs were detected in chr21 (Table S4). Based on a manual review using IGV, four SVs were removed (Fig. S6). The remaining 15 SVs were considered promising candidates, and all were located in the oscillated region (Table S5). Of the 15 SVs, 10 SVs were found in our previous study[1], and five were newly identified by the long reads (SV 6, 7, 9, 13, and 15). All breakpoints of the SVs were at the boundaries of the copy number blocks and in intergenic or intronic regions.

*Reconstruction of breakpoint sequences*

To reveal the DNA sequences of the SV breakpoints, we performed a *de novo* local

assembly of reads spanning each SV breakpoint using LoMA software[2]. Consensus sequences with SVs were successfully generated for 13 out of the 15 SVs and showed aberrant connections of the copy number blocks (Fig. S9). The consensus sequences of SV-ID 5, 6, 7, and 9 indicated that these SVs were close to one another and considered to compose one SV cluster (Fig. S9E). The consensus sequence with SV-ID 13 clearly revealed the connection of blocks M and S with a 2,710 bp insertion (Fig. S9J). Although the assemblies of SV-ID 1 and SV-ID 15 failed to generate consensus sequences with SVs, IGV visualization indicated the presence of breakpoints (Fig. S10), and the corresponding reads were consistently aligned with the SVs.

*Validation of SVs and consensus sequences*

Because the five novel SVs (ID 6, 7, 9, 13, 15) were not identified in our previous study, we experimentally validated them by PCR and Sanger sequencing (Fig. S8). Sanger sequencing of SV-ID 6, 7, and 9 determined a 634 bp sequence of the PCR amplicon, which perfectly matches the consensus sequence generated by LoMA (Fig. S8A). For SV-ID 13, Sanger sequencing determined 873 bp and 1,370 bp sequences from the left and right primers, respectively, which perfectly matches the consensus sequence (Fig. S8B); however, the DNA sequence of the intermediate regions could not be determined due to a repetitive sequence. Sanger sequencing of SV-ID 15 determined a 925 bp sequence, which is consistent with the mapping pattern of the reads (Fig. S8C). These results suggest the accuracy of our SV calling and the assembly using LoMA.

*Patient-specific transcripts detected by long-read RNA-seq*

We further sequenced RNA of the patient and her mother using long-read sequencing (Table S7). SPLICE software identified three patient-specific transcripts[8] (Table S8); two of them are known transcripts of *RNA binding motif protein 11* (*RBM11*:ENST00000400577.4 and ENST00000468643.5), a protein-coding gene located in the oscillated region; the third transcript was a novel transcript of *ATP binding cassette subfamily C member 13* (*ABCC13*), a pseudogene also located in the oscillated region.

*Promoter methylation of overexpressed genes*

An analysis of the RNA-seq data demonstrated that the maternal alleles of the 35 genes in the patient's chr21 were overexpressed (Fig. S14, Table S6). We examined DNA methylation of the maternal and paternal reads in the promoters of 11 protein-coding genes that were located in the oscillated region. Although 649 CpGs were in the promoter regions of the 35 genes, only seven CpGs showed different methylation rates (Table S13):

three out of 187 CpGs in *Ubiquitin specific peptidase* (*USP25*) promoter, three out of 95 CpGs in *CXADR Ig-Like Cell Adhesion Molecule* (*CXADR*), and one out of 76 CpGs in *MIR155 Host Gene* (*MIR155HG*). These results suggest that the methylation of promoters has no effect on the regulation of gene expression, at least in peripheral blood leukocytes.

*Limitation of this study*

Although our comprehensive study of the patient with tetrasomy 21 revealed the genomic structure, transcriptome, and DNA methylation status of the patient's extra chr21, our study still has several limitations. First, the exact order of the six-copy regions and the four-copy regions remains elusive. Ultra-long read sequencing may reveal the true structure[9]. Second, the structural analysis of 21p was insufficient due to the technical difficulties of analyzing highly repetitive regions. Third, although our analysis detected an upregulation of genes related to development, their association with the patient's phenotype is unclear. Fourth, experimental validation is required for confirming our hypothesis that one of the two centromeres is inactivated by DNA methylation. Despite these limitations, our analyses using long-read sequencing technologies and novel bioinformatics methods provides important information on the mechanisms of the structural changes and the biological impact of a complex chromosome abnormality.

**Reference**

1. Takano T, Nakabayashi K, Ota H, Arai Y, Kamura H, Hata K. Tetrasomy 21 pter→q21.3 due to an extra +dic(21;21)mat in a severely psychomotor-retarded female patient without Down syndrome phenotype. Eur J Med Genet. 2020;63(4):103824. doi:10.1016/j.ejmg.2019.103824
2. Ikemoto K, Fujimoto H, Fujimoto A (2023) Localized assembly for long reads enables genome-wide analysis of repetitive regions at single-base resolution in human genomes. Hum Genomics 17:21. https://doi.org/10.1186/s40246-023-00467-7
3. Li H, Durbin R. Fast and accurate short read alignment with Burrows–Wheeler transform. Bioinformatics. 2009;25(14):1754-1760. doi:10.1093/bioinformatics/btp324
4. McKenna A, Hanna M, Banks E, et al. The Genome Analysis Toolkit: A MapReduce framework for analyzing next-generation DNA sequencing data. Genome Res. 2010;20(9):1297-1303. doi:10.1101/gr.107524.110
5. Kim D, Langmead B, Salzberg SL. HISAT: a fast spliced aligner with low memory requirements. Nat Methods. 2015;12(4):357-360. doi:10.1038/nmeth.3317
6. Liao Y, Smyth GK, Shi W. featureCounts: an efficient general purpose program for

assigning sequence reads to genomic features. Bioinformatics. 2014;30(7):923-930. doi:10.1093/bioinformatics/btt656

7. Hart T, Komori HK, LaMere S, Podshivalova K, Salomon DR. Finding the active genes in deep RNA-seq gene expression studies. BMC Genomics. 2013;14(1). doi:10.1186/1471-2164-14-778
8. Kiyose H, Nakagawa H, Ono A, et al. Comprehensive analysis of full-length transcripts reveals novel splicing abnormalities and oncogenic transcripts in liver cancer. PLoS Genet. 2022;18(8):e1010342. doi:10.1371/journal.pgen.1010342
9. Jain M, Koren S, Miga KH, et al. Nanopore sequencing and assembly of a human genome with ultra-long reads. Nat Biotechnol. 2018;36(4):338-345. doi:10.1038/nbt.4060

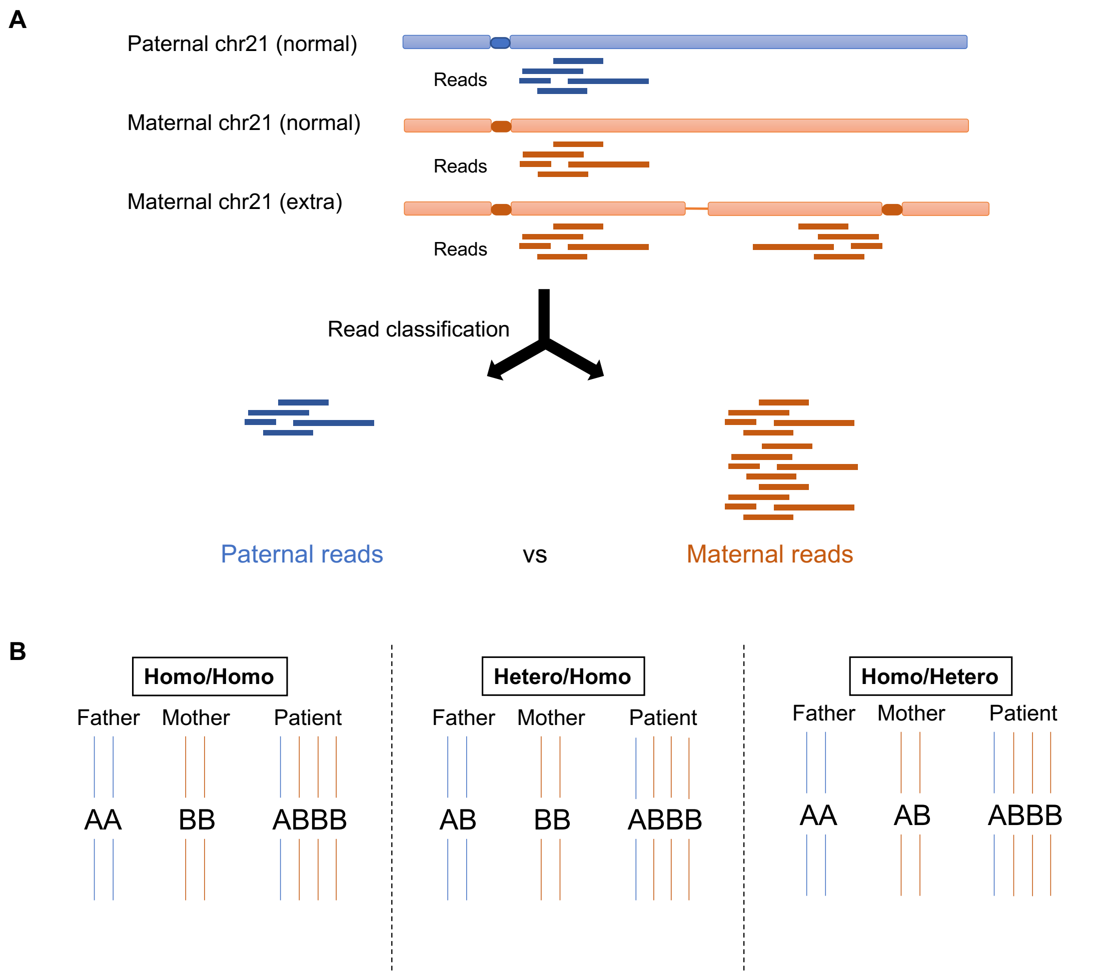


**Fig. S1 Estimation of allele origins from informative SNVs**

A: Schematic figure of the read classification. Our analyses addressed the abnormalities of extra chr21, which was maternal. Classification of the chromosomal origin of the reads enabled us to compare the normal paternal chromosome and the maternal chromosomes, which include extra chr21.

B: Three patterns of the parents' zygosity that allow estimation of the allele origins of the patient's SNVs are shown. The patterns are shown in bold text in the boxes in the order of the father's zygosity and the mother's zygosity. Theoretically, in these patterns, the patient's A allele would be derived from the father, and B allele would be derived from the mother. These informative SVs were used to classify the patient's reads by origin. Reads of the patient were classified as paternal or maternal in origin when an allele of informative SNVs on the reads was only carried by one parent. When both parents carried the allele, the read was considered indistinguishable.

**A**

| Father/Mother | Father | Mother | Patient |
|---|---|---|---|
| Homo/Homo | AA | BB | AB |

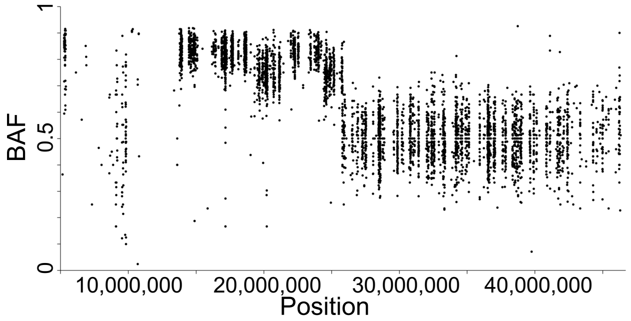


**B**

| Father/Mother | Father | Mother | Patient |
|---|---|---|---|
| Hetero/Homo | AB | BB | AB |

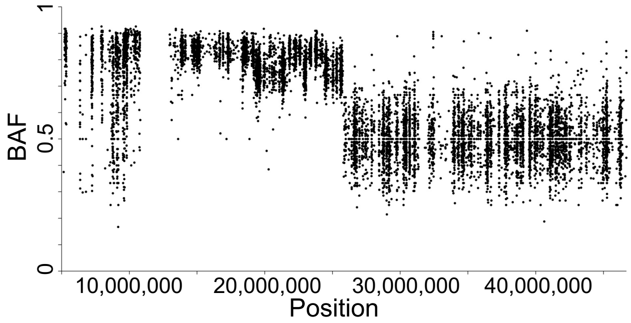


**C**

| Father/Mother | Father | Mother | Patient |
|---|---|---|---|
| Homo/Hetero | AA | AB | AB |

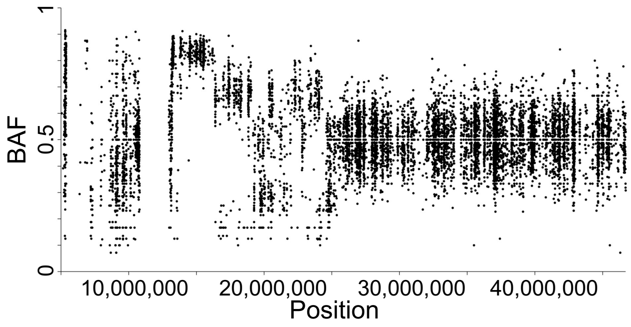


**Fig. S2 B allele frequency (BAF) of informative SNVs in the patient's chr21**

B allele represents the allele that was estimated as maternal (see Fig. S1B). SNVs were classified by the three patterns of the parents' zygosity.

A: BAF of SNVs that were homozygous in both parents.

B: BAF of SNVs that were heterozygous in the father and homozygous in the mother.

C: BAF of SNVs that were homozygous in the father and heterozygous in the mother.

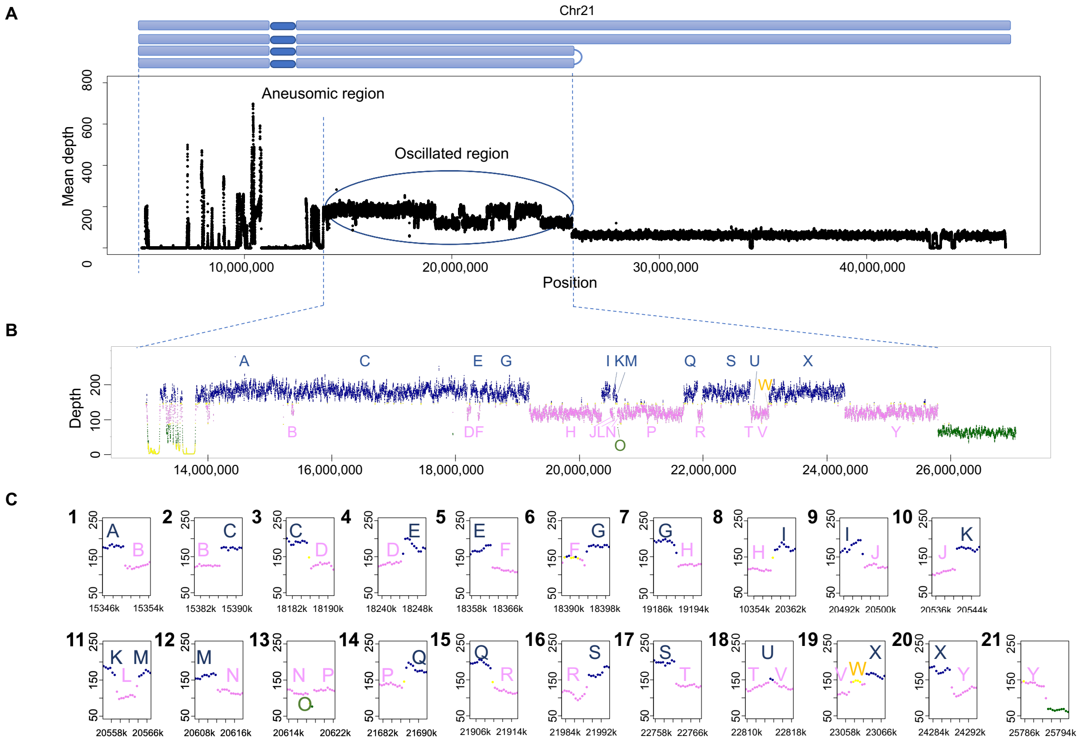


**Fig. S3 Depth of coverage of chr21 detected in gDNA long-read sequencing of the patient**

A: The mean depth of coverage for reads with a mapping quality ≥ 60 within 500 bp bins. The copy number oscillation was observed in 21q ("oscillated region").

B: The mean depth of coverage in the oscillated region. Green, pink, and blue indicate, respectively, from 31.6 to 85.26 (two-copy regions), from 90.05 to 143.69 (four-copy regions), and from 148.49 to 202.13 (six-copy regions). Other values are shown in yellow.

C: The boundaries of the oscillation. Each letter represents a copy number block (> 1 kb) in the oscillated region. Denoted in green, pink, yellow, and blue are two-copy regions, four-copy regions, five-copy region, and six-copy regions, respectively.

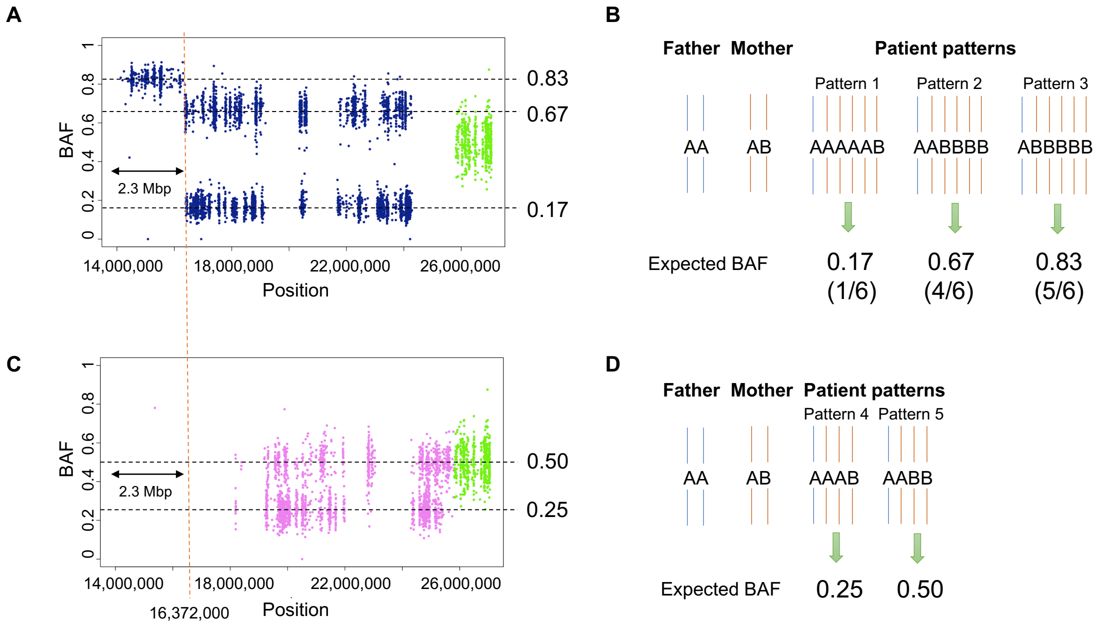


**Fig. S4 Differences of the patient's BAF in the six-copy and four-copy regions**

A: Pattern of B allele frequency (BAF) in the six-copy region. Blue and green indicate six-copy and two-copy regions, respectively.

B: Possible patterns of the patient's genotype based on the BAF distribution.

C: Pattern of BAF in the four-copy region. Pink and green indicate four-copy and two-copy regions, respectively.

D: Possible patterns of the patient's genotype based on the BAF distribution.

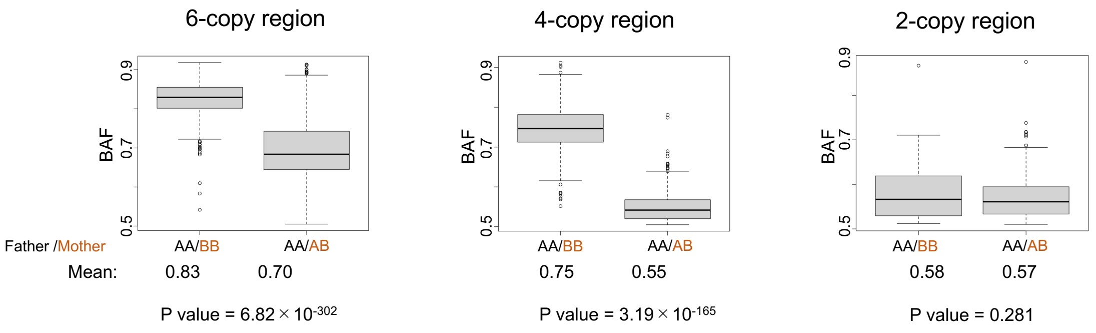


**Fig. S5 Differences in patient's BAF between SNVs that were homozygous and heterozygous in the mother's genotype**

Box plots show the BAF distribution for SNVs whose zygosity pattern of the parents were AA/BB and AA/AB. BAF was significantly low in SNVs of the AA/AB pattern in six-copy and four-copy regions, but no significant difference was found in two-copy regions. P values were obtained using Wilcoxon's rank sum test.

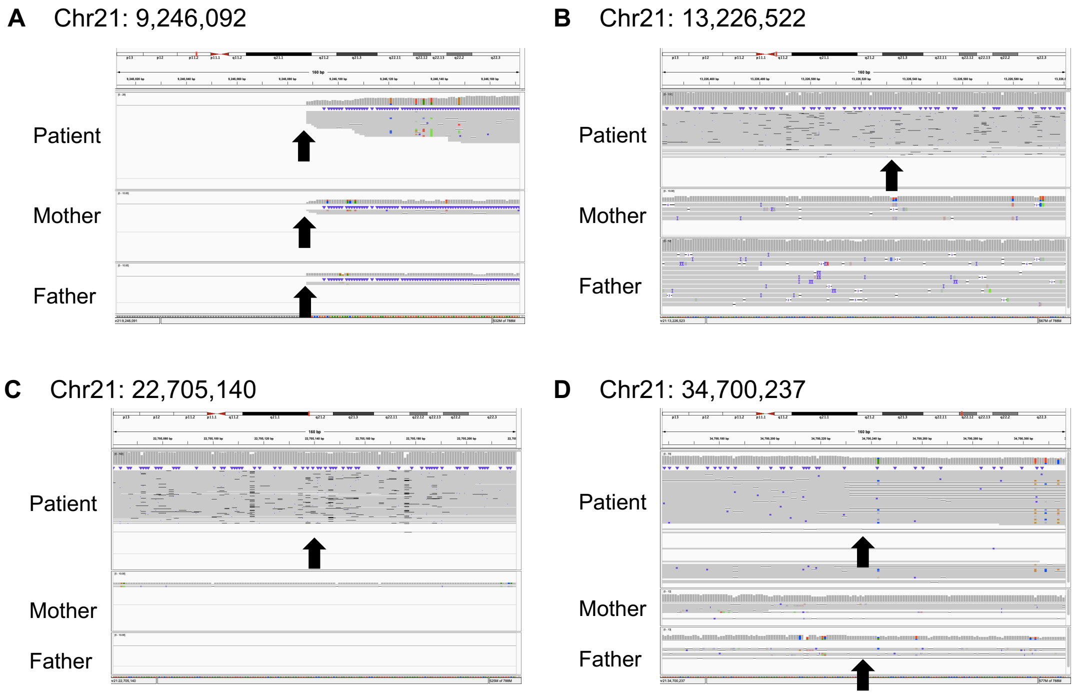


**Fig. S6 IGV visualization of the SVs removed from the analyses**

Patient-specific SVs (Table S4) were visually confirmed with IGV, and the following four SVs were removed from the analyses for the reasons below.

A: A chromosomal translocation between chr21: 9,246,092 and chr22_KI270736v1_random. An identical breakpoint was found in both parents.

B: A chromosomal translocation between chr17: 22,153,701 and chr21: 13,226,522. No breakpoint was found.

C: An insertion of >20,012 bp in chr21:22,705,140. No breakpoint was found.

D: A deletion of 855 bp in chr21:34,700,237-34,701,093. The deletion was also found in the father.

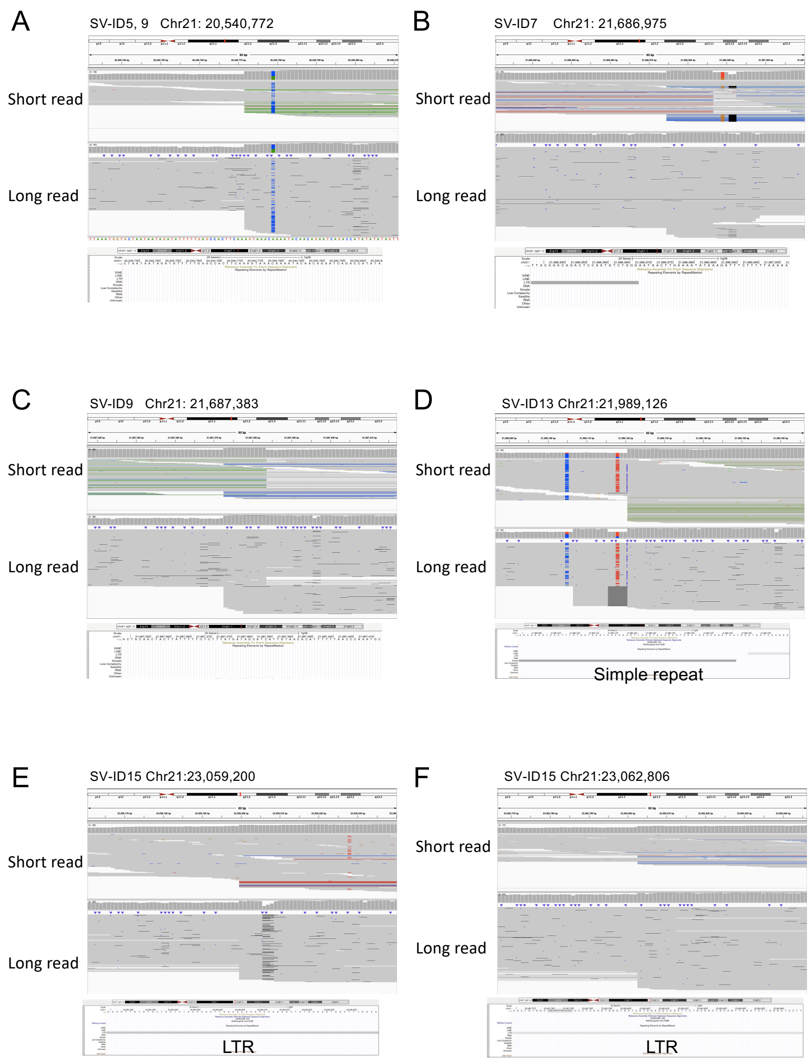


**Fig. S7 Visualization of short reads and long reads using IGV for the novel SVs**

Clear breakpoints were observed in both long and short reads. These breakpoints were located within repeat regions, which could potentially lead to false negatives in short reads.

A: IGV visualization of SV-ID5 and SV-ID9, B: IGV visualization of SV-ID7, C: IGV visualization of one breakpoint of SV-ID9, D: IGV visualization of SV-ID13, E: IGV visualization of one breakpoint of SV-ID15, F: IGV visualization of one breakpoint of SV-ID15.

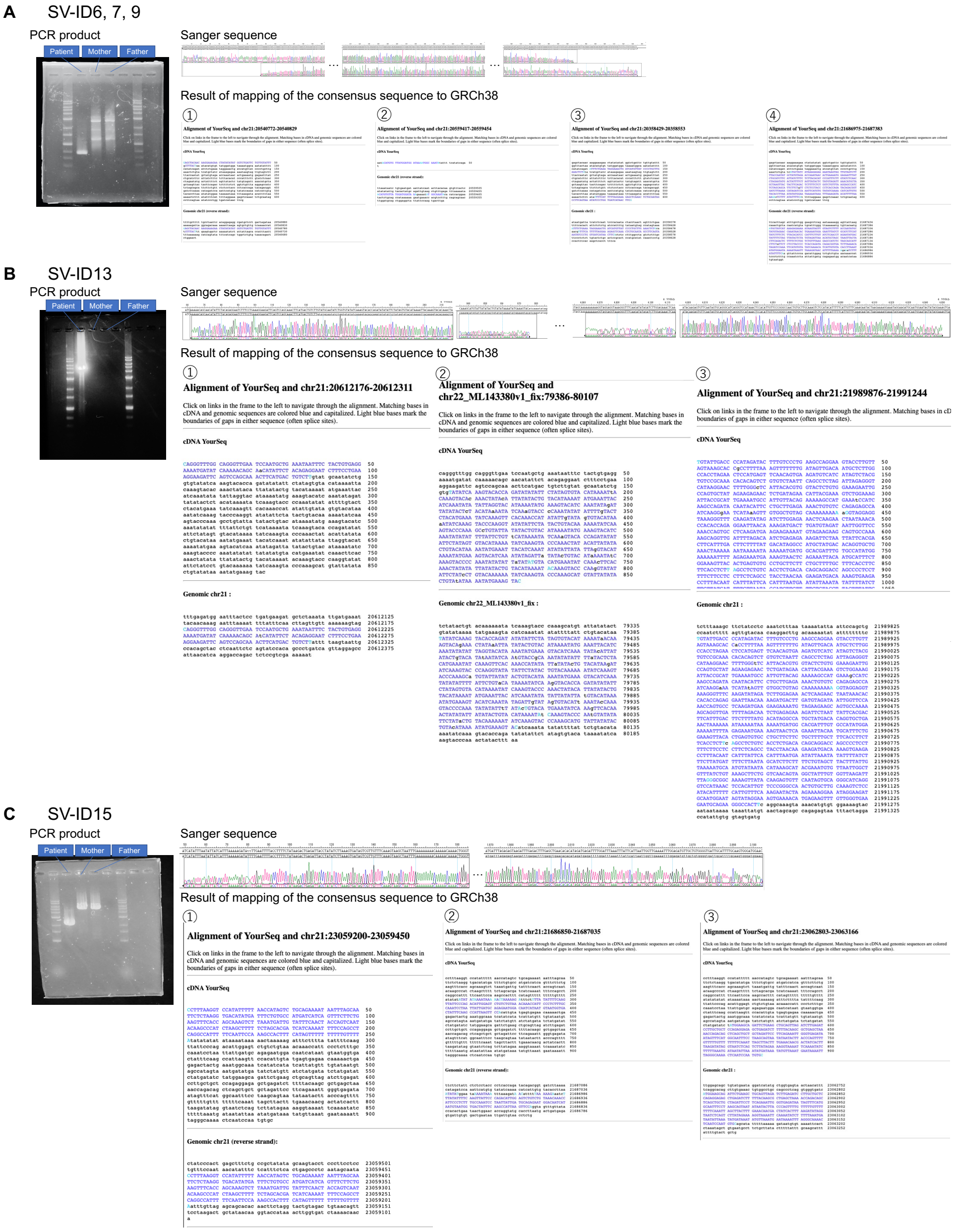


**Fig. S8 Validation of SVs that were identified only by long-read sequencing**

Gel electrophoresis images of the PCR products, in which the patient's specific bands with expected size were detected only in the patient's lane; Sanger sequencing electropherograms of the patient's specific bands; and mapping results of the sequence using web-BLAT (https://genome.ucsc.edu/cgi-bin/hgBlat) are shown. Consensus sequences generated by Sanger sequencing were mapped to the reference genome. Aligned bases are in blue, and unaligned bases are in black.

A–C: SV-ID 6, 7, 9 (A), SV-ID 13 (B), SV-ID 15 (C).

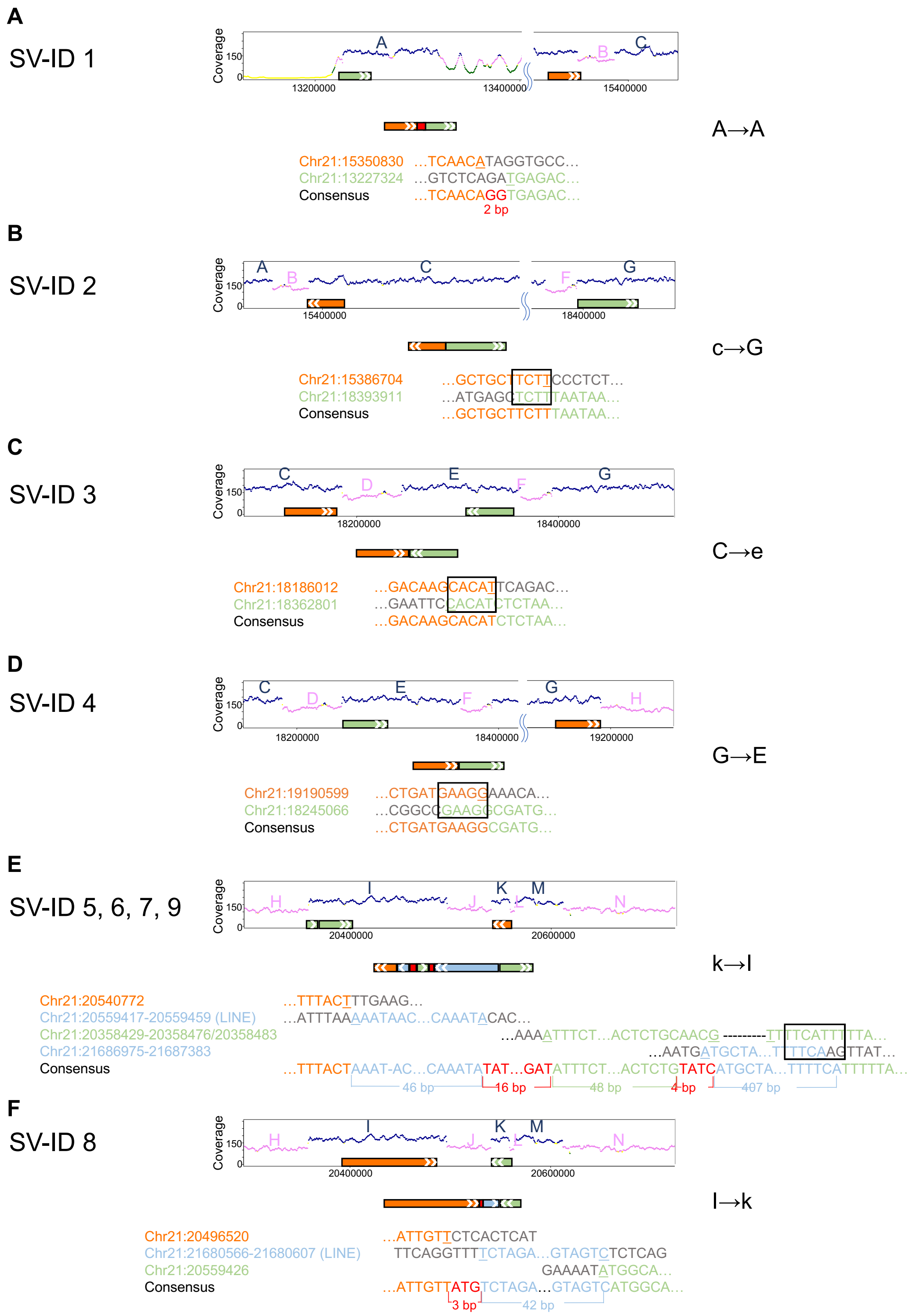


**Fig. S9 Schematic overview of the alignment of irregular sequences estimated from local assemblies (1/2)**

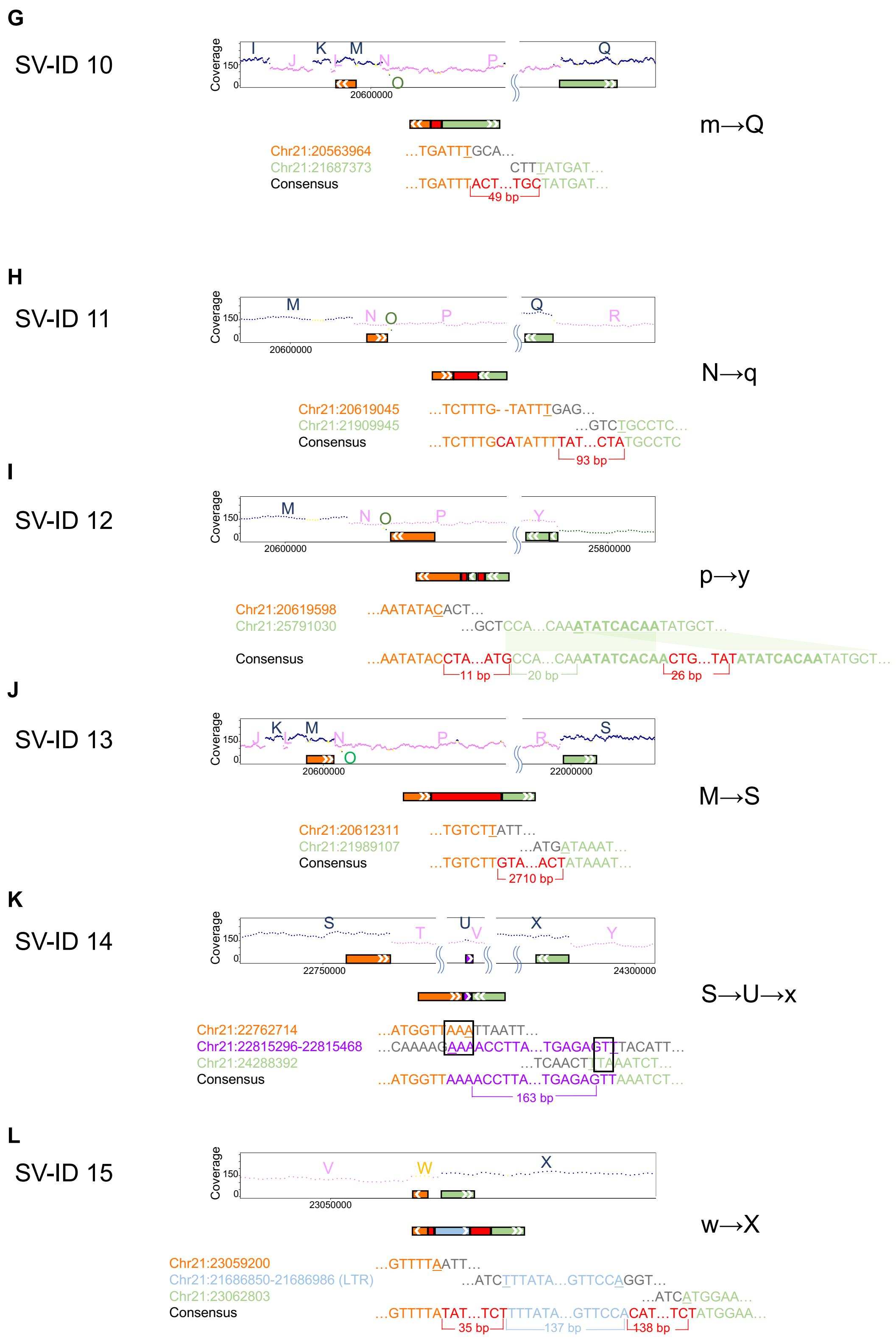


**Fig. S9 Schematic overview of the alignment of irregular sequences estimated from local assemblies (2/2)**

Alignments of consensus sequences with aberrant rearrangements are shown. Each plot shows the coverage of the copy number blocks involved in SVs (see Fig. 1). Rectangles in orange, green, and purple denote the loci of the consensus sequences in the reference genome and the arranged order of the patient's extra chr21. Their orientations are shown by arrows in the rectangles. Rectangles in light blue and red are insertions of known and unknown sequences. Nucleotide sequences show the reference genome and the consensus sequences spanning the breakpoints. Sequence colors correspond to the rectangles, and unmatched references are colored in gray. Homologies between two blocks are framed with black rectangles. The positions indicate the chromosomal locations of the underlined nucleotides in the reference genome. The consensus sequence of SV-ID 1 represents a sequence of the corresponding read, the consensus sequences of SV-ID 2 to 14 were generated by LoMA, and the consensus sequence of SV-ID 15 was generated by Sanger sequencing. +, plus strand; –, minus strand.

A: Alignment of the consensus sequence of SV-ID 1 shows a tandem duplication of block A (+). Because no insertion nor homology was found at the junction, the connection might be the result of non-homologous end joining (NHEJ).

B: Alignment of the consensus sequence of SV-ID 2 shows a connection of block C (–) and block G (+). Because a 4-bp homology was found at the junction, these blocks might have been connected by alternative end joining (alt-EJ).

C: Alignment of the consensus sequence of SV-ID 3 shows a connection of block C (+) and block E (–). Because a 5-bp homology was found at the junction, the most plausible mechanism is alt-EJ.

D: Alignment of the consensus sequence of SV-ID 4 shows a connection of block G (+) and block E (+). Because a 5-bp homology was found at the junction, the most plausible mechanism is alt-EJ.

E: Alignment of the consensus sequence of SV-ID 5, 6, 7, 9 shows a connection of block K (–) and block I (+). Because an insertion of >10 bp was found, the most plausible mechanism is fork stalling and template switching or microhomology mediated break induced repair (FoSTeS/MMBIR).

F: Alignment of the consensus sequence of SV-ID 8 shows a connection of block I (+) and block K (–). Because an insertion of >10 bp was found, the most plausible mechanism is FoSTeS/MMBIR.

G: Alignment of the consensus sequence of SV-ID 10 shows a connection of block M (–) and block Q (+). Because an insertion of >10 bp was found, the most plausible mechanism is FoSTeS/MMBIR.

H: Alignment of the consensus sequence of SV-ID 11 shows a connection of block N (+)

and block Q (–). Because an insertion of >10 bp was found, the most plausible mechanism is FoSTeS/MMBIR.

I: Alignment of the consensus sequence of SV-ID 12 shows a connection of block P (–) and block Y (–). Because an insertion of >10 bp was found, the most plausible mechanism is FoSTeS/MMBIR.

J: Alignment of the consensus sequence of SV-ID 13 shows a connection of block M (+) and block S (+). Because an insertion of >10 bp was found, the most plausible mechanism is FoSTeS/MMBIR.

K: Alignment of the consensus sequence of SV-ID 14 shows a connection of block S (+), block U (+), and block X (–). Because 3-bp and 2-bp homologies were found, the most plausible mechanism is alt-EJ.

L: Alignment of the consensus sequence of SV-ID 15 shows a connection of block W (–) and block X (+). Because an insertion of >10 bp was found, the most plausible mechanism is FoSTeS/MMBIR.

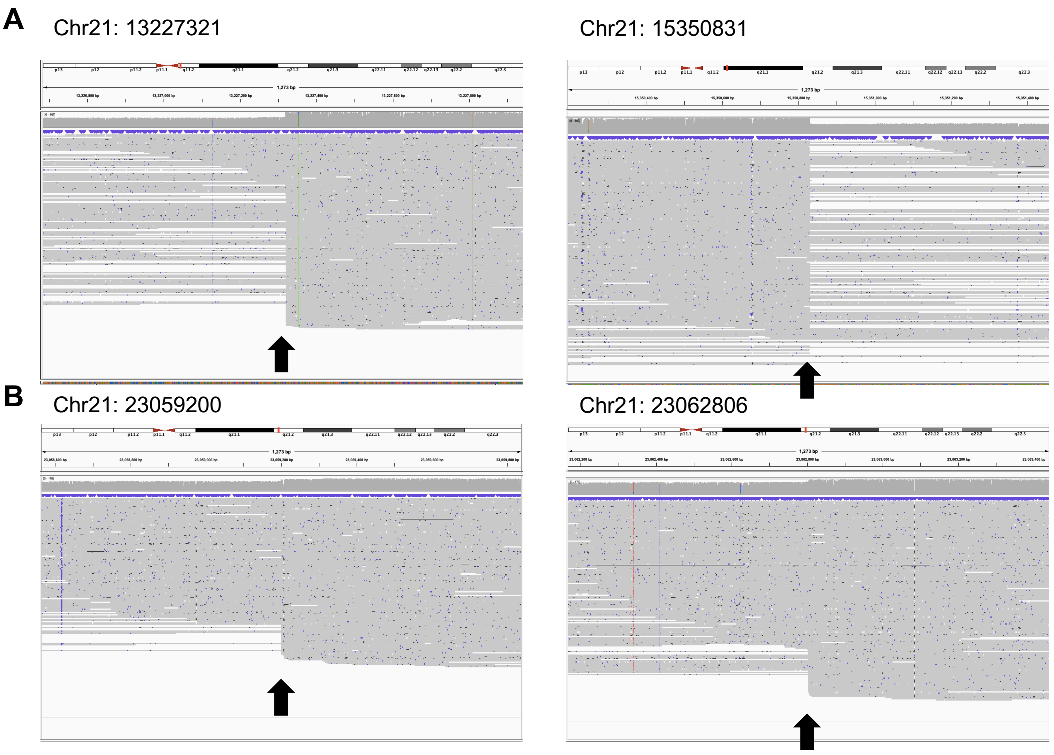


**Fig. S10 IGV visualization of the SV breakpoints that failed to generate a consensus sequence**

A: Two breakpoints of SV-ID 1, chr21: 13,227,321 (left) and chr21:15,350,831 (right).

B: Two breakpoints of SV-ID 15, chr21: 23,059,200 (left) and chr21: 23,062,806 (right).

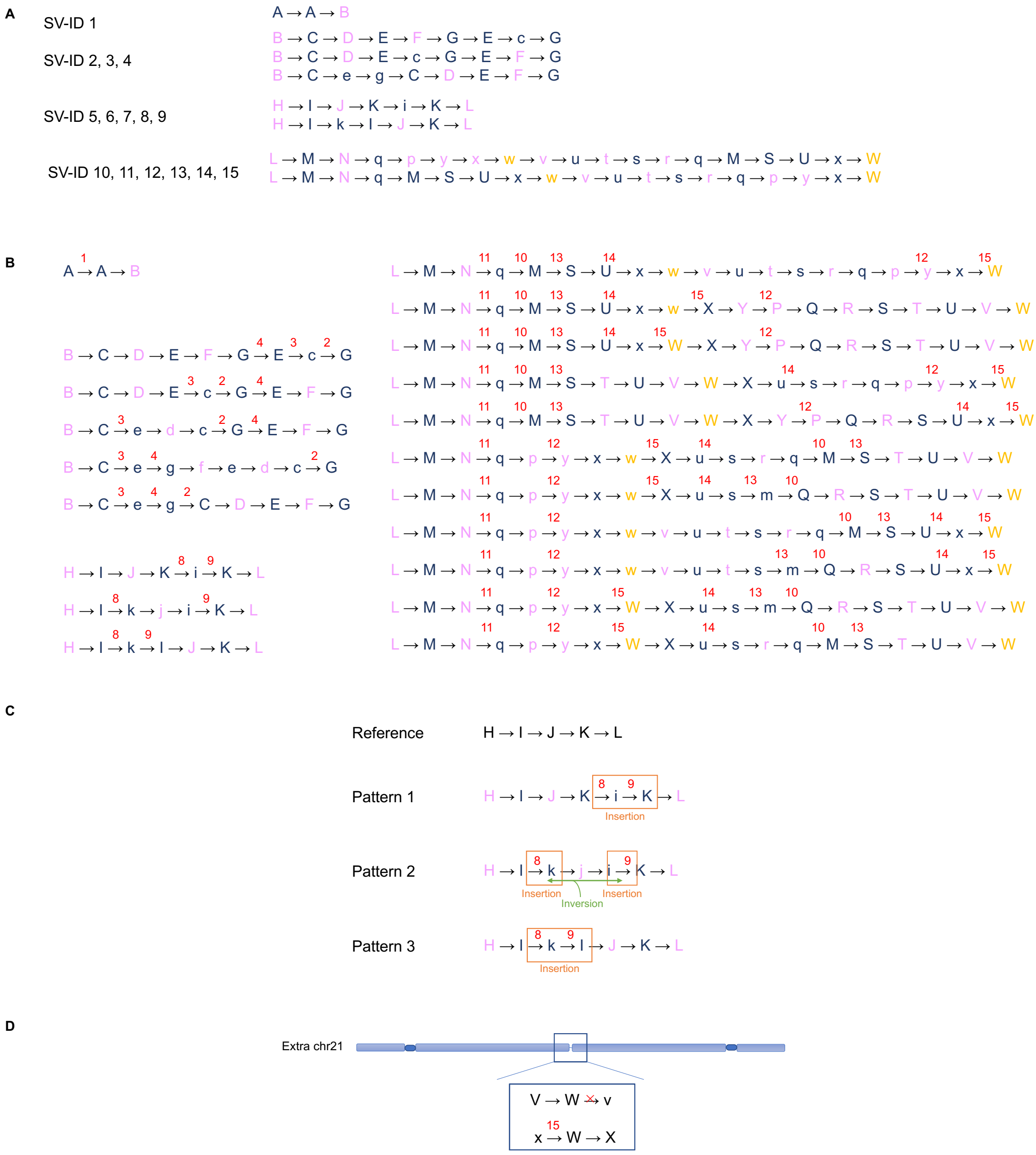


**Fig. S11 Possible orders of regions in the oscillated region of extra chr21**

A: Possible orders of rearranged blocks estimated from the consensus sequences of candidate SVs. Letters correspond to Fig 1. Denoted in pink, yellow, and blue are four-copy regions, the five-copy region, and six-copy regions, respectively. Upper and lower cases represent forward and reverse strands, respectively. Patterns including minimum events were selected from all possible patterns.

B: All possible orders of the rearranged regions. Letters correspond to those in Fig. 1. Pink, yellow, and blue: four-copy regions, the five-copy region, and six-copy regions, respectively. Upper and lower cases represent forward and reverse strands, respectively.

Numbers in red above the arrows are SD-IDs corresponding to Table S5, which indicates aberrant connections of regions. Clustered SV-ID 5, 6, 7, 9 is denoted as “9”. Arrows without numbers are connections that are the same as the reference genome.

C: Patterns 1, 2, and 3 show the three possible patterns of the region including blocks H, I, J, K, and L. The order of the reference genome is shown for comparison. Structural changes compared with the reference genome are marked in orange and yellow.

D: Two possible patterns of regions in the middle of extra chr21. Upstream of block W could be block V as a reference genome or block X (– strand) as SV-ID 15. However, the former is unreasonable considering the other side of symmetry and blocks W and V (– strand) have no connection. The latter is feasible, because blocks W and X are successive in the reference genome.

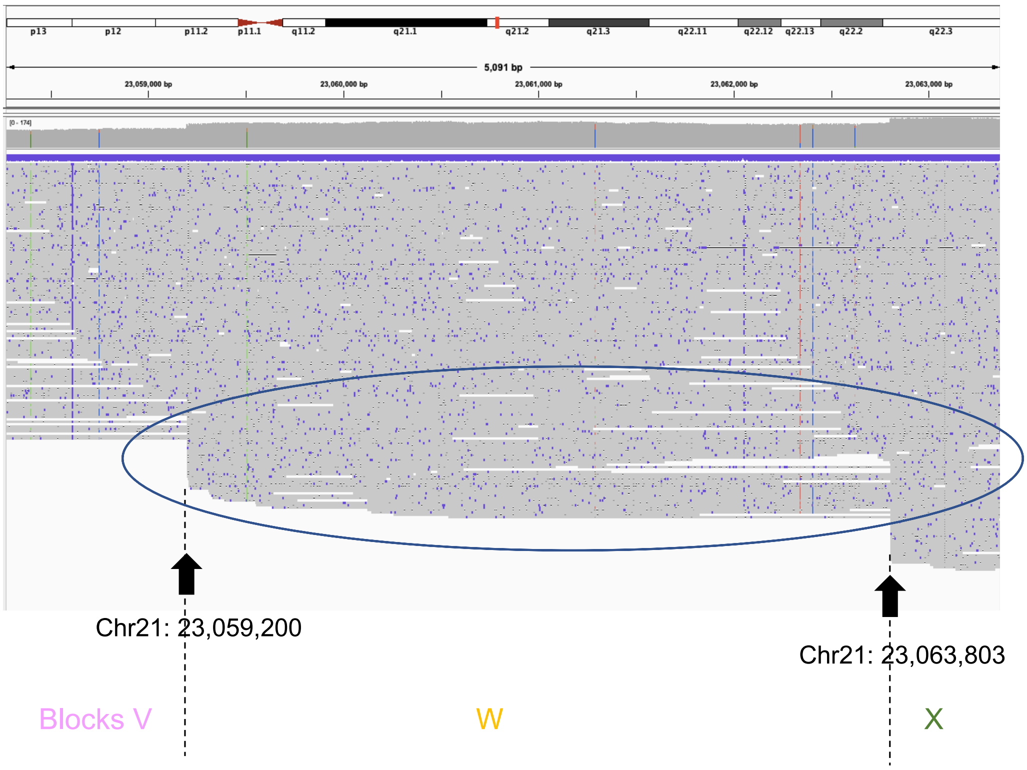


**Fig. S12 IGV visualization of block W**

Breakpoints of SV-ID 15 are related to the boundaries of blocks V and W and blocks W and X. Some reads marked in the oval are aligned with breakpoints in chr21:23,590,200 that connect with block X (– strand) on the left side and span blocks W and X on the right side.

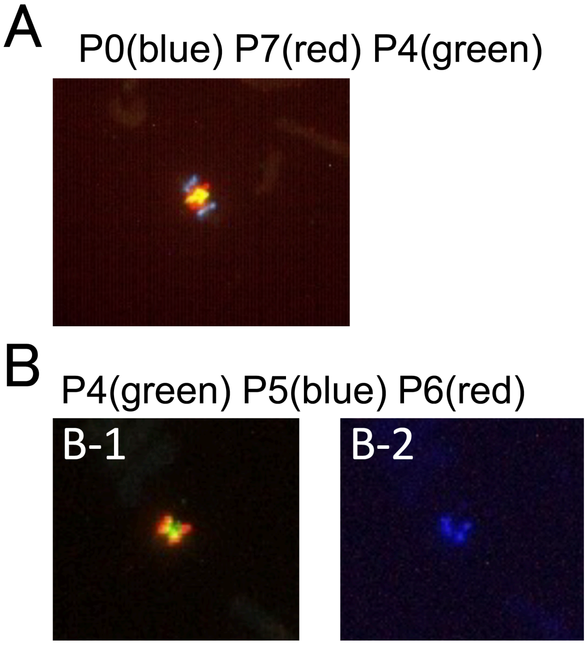


**Fig. S13** FISH using P0, P4, P5, P6 and P7

A: FISH using P0, P4 and P7. Blue P0 signals near the centromere are located outmost, followed by the red P7 signals. The yellow signals are the region where P4 and P7 appear to overlap.

B: FISH using P4, P5 and P6. Green P4 signals are inside or close to the fused region without a gap. In addition to the clearly visible red P6 signals, yellowish P6 signals overlapping P4 and P5 are observed (B-1). Blue P5 signals are close to the position of the red P6 signals (B-2).

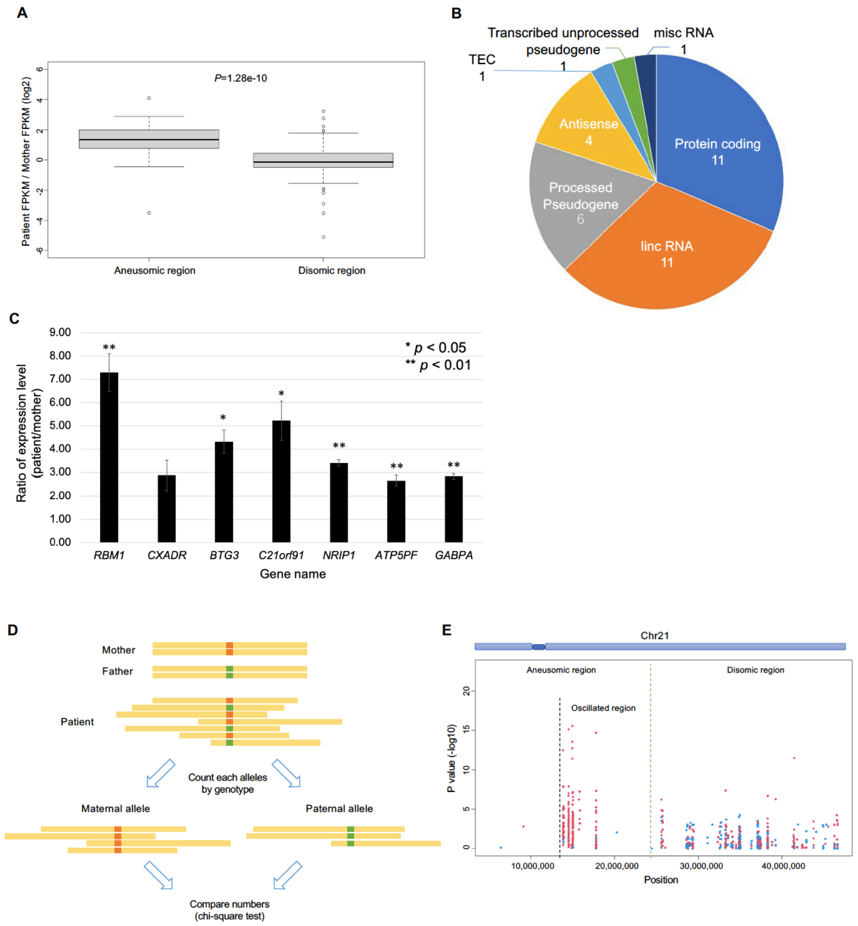


**Fig. S14 Overexpression and allelic imbalance of the aneusomic region detected by RNA-seq analysis**

A: Distribution of the Patient-to-Mother FPKM ratio in chr21. The aneusomic region had a significantly higher ratio than the disomic region. *P* values were obtained using the Wilcoxon rank sum test.

B: Breakdown of the 35 genes expressed in the oscillated region.

C: qPCR of seven genes. All genes showed higher expression levels in the patient compared to her mother, with six of the genes demonstrating statistical significance. (For *CXADR* gene, $p = 0.053$).

D: A schematic overview of the haplotype classification and statistical test.

E: P values from chi-square tests comparing the distribution of maternal reads and paternal reads with expected values. Red dots indicate the number of maternal reads is larger than that of paternal reads. A peak *P* value was observed in the oscillated region. After the Bonferroni correction, 67 loci remained significant.

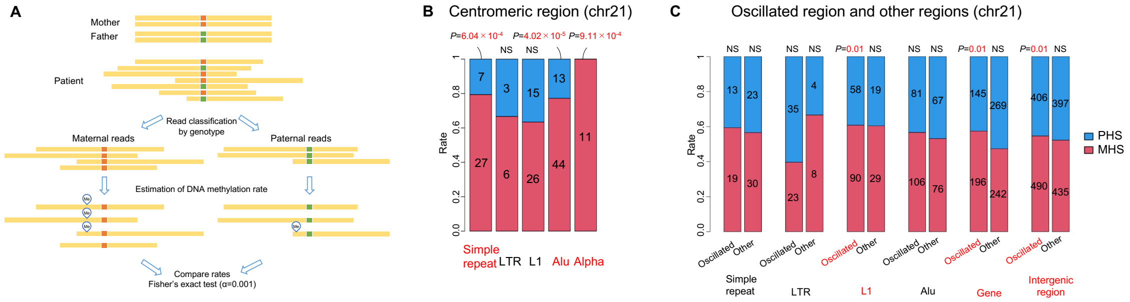


**Fig. S15 Hypermethylation of maternal reads compared to paternal reads in the centromere and the oscillated region of chr21**

A: A schematic overview of the read classification and statistical test.

B: Rates of maternally and paternally hypermethylated CpG sites in the chr21 centromeric region. *P* values were obtained using the chi-square test. NS, not significant.

C: Rates of maternally and paternally hypermethylated CpG sites in the chr21 oscillated region and other regions. *P* values were obtained using the chi-square test. NS, not significant.

Cent, centromeric region; Oscillated, oscillated region; Other, other regions; LTR, long terminal repeats; L1, L1 transposon; Alu, *Alu* element; Alpha, alpha satellite repeat.

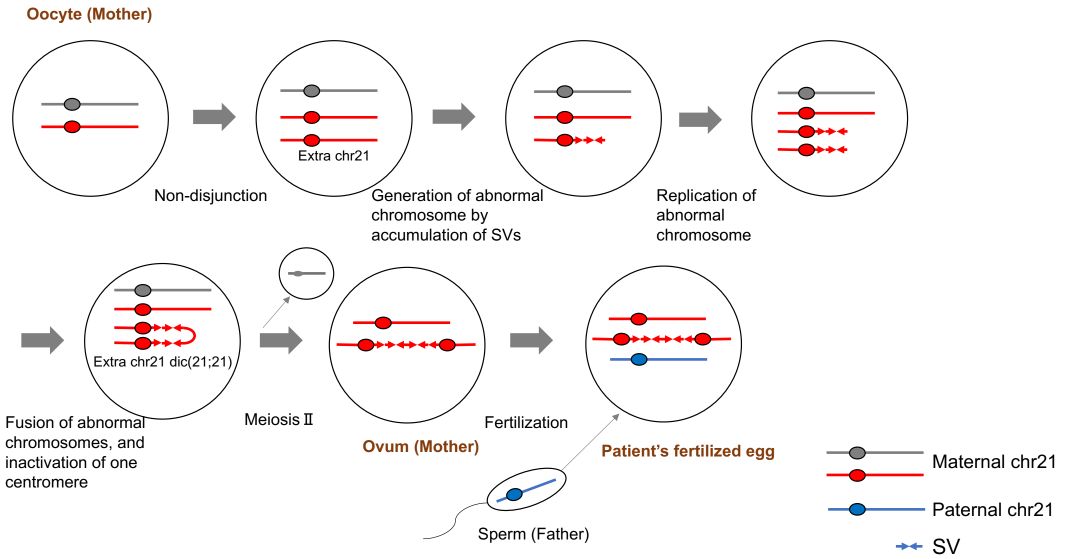


**Fig. S16 A model of the formation of the extra chr21**

A schematic model of the possible sequential events that result in the generation of extra chr21 in the patient during oogenesis in the patient's mother.
