# Supplementary material for: Long-read sequencing reveals the complex structure of extra dic(21;21) chromosome and its biological effects
Source: Hum Genet. 2023 Jul 11;142(9):1375–84. doi: 10.1007/s00439-023-02583-9 (PMC10449678; doi:10.1007/s00439-023-02583-9)
Supplement: Supplementary file 2 — Supplementary file2 (PDF 379 KB) [file 439_2023_2583_MOESM2_ESM.pdf]

Supplementary Tables

Long-read sequencing reveals the complex structure of extra dic(21;21) chromosome and its biological effects

Kugui Yoshida-Tanaka[1], Ko Ikemoto[1], Ryoji Kuribayashi[1], Motoko Unoki[1], Takako Takano[2,3], and Akihiro Fujimoto[1]

1 Department of Human Genetics, School of International Health, Graduate School of Medicine, The University of Tokyo, Tokyo, Japan

2 Department of Child Health, Tokyo Kasei University, Tokyo, Japan

3 Tokyo Metropolitan Tobu Medical Center for Children with Developmental Disabilities, Tokyo, Japan

Corresponding author:

Takako Takano

Department of Child Health, Tokyo Kasei University

1-18-1 Kaga, Itabashi-ku, Tokyo 173-8602, JAPAN

TEL & FAX: +81-3-3961-5339

E-mail: takano@tokyo-kasei.ac.jp

Akihiro Fujimoto

Department of Human Genetics, The University of Tokyo, Graduate School of Medicine,

7-3-1 Hongo, Bunkyo-ku, Tokyo, 113-0033, JAPAN

TEL: +81-3-5841-3692

E-mail: afujimoto@m.u-tokyo.ac.jp

Table S1. Probes used in FISH (GRCh38)

Name of probe	Targeted to Chr21 (GRCh38)	Size (kbp)	Dye	Color	Oscillated copy numbers of dic(21;21) *	Block
P0 (enumeration)	chr21:14,125,858–14,778,973	653	aqua	blue	6 copies	A
G0 (enumeration)	chr21:14,125,858–14,778,973	653	FITC	green	6 copies	A
P1 (our designated)	chr21:19,665,945–19,821,775	156	Cy3	red	4 copies	H
P2 (our designated)	chr21:24,897,941–25,051,098	151	FITC	green	4 copies	Y
P3 (our designated)	chr21:22,077,681–22,227,681	150	Cy3	red	6 copies	S
P4 (our designated)	chr21:23,527,681–23,677,682	150	FITC	green	6 copies	X
P5 (our designated)	chr21:18,627,682–18,777,682	150	aqua	blue	6 copies	G
P6 (our designated)	chr21:15,387,000–15,537,000	150	Cy3	red	6 copies	C
P7 (our designated)	chr21:21,760,000–21,910,000	150	Cy3	red	6 copies	Q

* see Fig. 1A; FITC, fluorescein isothiocyanate

Table S2. Summary of long-read whole genome sequencing

Run	Samples	Yield (Gbp)	Number of reads	Mean read length (bp)
1	Patient	38.7	7,919,982	4,840
2	Patient	34.6	7,072,124	4,840
3	Patient	28	5,191,529	5,340
4	Patient	13.2	1,675,522	7,830
5	Patient	25.3	3,753,433	6,680
6	Patient	15.3	2,072,759	7,350
7	Patient	39.9	8,282,352	4,770
8	Mother	33.7	7,216,869	4,630
9	Father	34.4	7,355,235	4,630

Table S3. Location and copy number of each block

Block	Start position (chr 21)	End position (chr 21)	Copy number
A	13,000,000	15,351,000	6
B	15,351,001	15,386,000	4
C	15,386,001	18,186,000	6
D	18,186,001	18,244,500	4
E	18,244,501	18,363,000	6
F	18,363,001	18,393,500	4
G	18,393,501	19,190,500	6
H	19,190,501	20,358,000	4
I	20,358,001	20,496,500	6
J	20,496,501	20,540,500	4
K	20,540,501	20,559,500	6
L	20,559,501	20,563,500	4
M	20,563,501	20,612,500	6
N	20,612,501	20,619,000	4
O	20,619,001	20,620,000	2
P	20,620,001	21,686,500	4
Q	21,686,501	21,910,000	6
R	21,910,001	21,988,500	4
S	21,988,501	22,763,000	6
T	22,763,001	22,815,000	4
U	22,815,001	22,815,500	6
V	22,815,501	23,059,000	4
W	23,059,001	23,062,000	5
X	23,062,001	24,288,500	6
Y	24,288,501	25,790,500	4

Table S4. Specific structural variants detected in chr21 of the patient

Breakpoint 1		Breakpoint 2						
Chr	Position	Chromosome	Position	Read number	SV type	Length	VAF	Reliability
chr21	9,246,092	chr22_KI270736v1_random	175,703	36	CHR	NA	1	Low (found in parents)
chr21	13,,227,321	chr21	15,350,831	60	TRS	2,123,508	0.543	High
chr21	15,386704	chr21	18,393,913	54	INV	3,007,207	0.353	High
chr21	18,186,016	chr21	18,362,787	68	INV	176,789	0.456	High
chr21	18,245,069	chr21	19,190,600	54	TRS	945,531	0.422	High
chr21	20,358,429	chr21	20,540,773	41	INV	182,344	0.284	High
chr21	20,358,479	chr21	20,358,479	28	INS	398	0.196	High
chr21	20,358,484	chr21	21,686,978	7	INV	1,328,498	0.052	High
chr21	20,496,524	chr21	20,559,427	58	INV	62,906	0.407	High
chr21	20,540,778	chr21	21,687,384	7	TRS	1,146,740	0.045	High
chr21	20,563,963	chr21	21,687,374	50	INV	1,123,413	0.337	High
chr21	20,619,046	chr21	21,909,946	41	INV	1,290,916	0.357	High
chr21	20,619,599	chr21	25,791,031	52	TRS	5,170,024	0.860	High
chr21	21,989,126	chrUn_KI270333v1	2,619	18	CHR	NA	0.310	High
chr17	2,2153,701	chr21	13,226,522	28	CHR	NA	0.136	Low (no breakpoint)
chr21	22,705,140	chr21	22,705,140	6	INS	>20012	0.037	Low (no breakpoint)
chr21	22,762,715	chr21	24,288,455	52	INV	1525678	0.323	High
chr21	23,059,200	chr21	23,062,806	20	INV	3600	0.141	High
chr21	34,700,237	chr21	34,701,093	13	DEL	855	0.271	Low (found in father)

NA, not available; CHR, chromosomal translocation; TRS, intra-chromosomal translocation; INV, inversion; DEL, deletion; INS, insertion; VAF, variant allele frequency

Table S5. Candidate SVs and features of breakpoints

ID	SV type	Chrom	Position	Boundary	Gene	Repeat	Successful assembly	Features between breakpoints: Insertion	Features between breakpoints: Homology
1	TRS	chr21	13,227,321	Start of A	-	LTR		NA	NA
		chr21	15,350,831	A-B	-	LINE			
2	INV	chr21	15,386,704	B-C	*ENSG00000229425*/intron 5 of 5	LTR	Yes	-	4 bp
		chr21	18,393,913	F-G	*TMPRSS15*/intron 3 of 24	-	Yes		
3	INV	chr21	18,186,016	C-D	*CHODL*/intron 2 of 6	LTR	Yes	-	5 bp
		chr21	18,362,787	E-F	*TMPRSS15*/intron 7 of 24	SINE	Yes		
4	TRS	chr21	18,245,069	D-E	*CHODL*/intron 2 of 6	-	Yes	-	5 bp
		chr21	19,190,600	G-H	-	-	Yes		
5	INV	chr21	20,358,429	H-I	-	LINE	Yes	51 bp	-
		chr21	20,540,773	J-K	-	-	Yes		
6	INS	chr21	20,358,479	H-I	-	LINE	Yes	411 bp	6 bp
7	INV	chr21	20,358,484	H-I	-	LINE	Yes	-	
		chr21	21,686,978	P-Q	-	-	Yes		
8	INV	chr21	20,496,524	I-J	-	-	Yes	45 bp	-
		chr21	20,559,427	K-L	-	LINE	Yes		
9	TRS	chr21	20,540,778	J-K	-	-	Yes	103 bp	-
		chr21	21,687,384	P-Q	-	-	Yes		
10	INV	chr21	20,563,963	L-M	-	-	Yes	49 bp	-
		chr21	21,687,374	P-Q	-	-	Yes		
11	INV	chr21	20,619,046	N-O	-	LTR	Yes	93 bp	-
		chr21	21,909,946	Q-R	-	-	Yes		
12	TRS	chr21	20,619,599	O-P	-	-	Yes	11 bp/ 26 bp	-
		chr21	25,791,031	End of Y	-	-	Yes		
13	CHR	chr21	21,989,126	R-S	-	Simple	Yes	NA	NA
		chrUn_KI270333v1	2,619		-	Satellite			
14	INV	chr21	22,762,715	S-T	-	-	Yes	-	3 bp/2 bp
		chr21	24,288,455	X-Y	-	-	Yes		
15	INV	chr21	23,059,200	V-W	-	LTR		310 bp	-
		chr21	23,062,806	W-X	-	LTR			

CHR, chromosomal translocation; TRS, intra-chromosomal translocation; INV, inversion; INS, insertion; DEL, deletion; LTR, long terminal repeats

Table S6. Genes overexpressed in patient's blood

	FPKM			Position		
Gene	Patient	Mother	Ratio	Start	End	Copy number
RBM11	3.843	0.525	7.315	14,216,130	14,228,372	6
ABCC13	27.329	10.395	2.629	142,36,206	14,362,754	6
HSPA13	49.570	14.639	3.386	14,371,115	14,383,484	6
SAMSN1	32.623	12.953	2.518	144,85,228	14,658,821	6
AF127936.2	0.5493	0.032	17.061	147,46,762	14,753,863	6
POLR2CP1	0.794	0	Inf	14,757,588	14,758,352	6
AF127936.1	3.561	1.118	3.187	14,761,710	14,763,090	6
AF127577.4	2.017	1.141	1.767	14,818,843	15,014,430	6
LINC02246	0.392	0	Inf	14,819,699	14,918,552	6
AF127577.3	0.354	0	Inf	14,918,534	14,947,096	6
NRIP1	29.743	11.717	2.538	14,961,235	15,065,903	6
AF127577.2	3.347	1.437	2.328	14,971,470	14,992,854	6
AF127577.6	5.974	1.592	3.753	15,050,189	15,052,379	6
RAD23BP3	0.729	0.183	3.986	15,694,300	15,696,581	6
USP25	92.140	29.539	3.119	15,730,025	15,878,911	6
RBPMSLP	4.297	1.727	2.488	15,744,004	15,745,080	6
CXADR	0.352	0.059	6.004	17,512,382	17,593,579	6
RF00019	0	0.481	0	17,527,140	17,527,247	6
BTG3	3.849	1.292	2.980	17,593,653	17,612,844	6
C21orf91-OT1	1.942	0.469	4.141	17,763,315	17,792,523	6
C21orf91	45.689	15.837	2.885	17,788,967	17,819,386	6
AL109761.1	8.878	2.227	3.987	17,793,488	17,810,845	6
CHODL-AS1	1.042	0.375	2.780	17,835,016	17,885,608	6
AF130417.1	0.782	0.220	3.557	18,022,370	18,114,904	6
PPIAP22	201.548	201.548	1	18,857,779	18,858,276	6
LINC02573	6.697	2.452	2.731	20,256,752	20,258,820	4
LINC01684	0.771	0.663	1.163	24,428,740	24,547,942	4
AP000233.1	0	0.359	0	25,127,469	25,135,247	4
RPL13AP7	3.180	3.304	0.962	25,361,821	25,362,431	4
MIR155HG	3.820	1.632	2.340	25,561,909	25,575,168	4
LINC00515	2.040	0.739	2.759	25,582,770	25,583,326	4
MRPL39	18.107	7.906	2.290	25,585,656	25,607,517	4
ATP5PF	10.396	4.523	2.298	25,716,503	25,735,639	4
GABPA	31.115	14.533	2.141	25,734,570	25,772,460	4
LLPHP2	1.180	0.780	1.512	25,762,938	25,763,333	4

Table S7. Summary of RNA-seq using long reads

Sample	Read number	Rate of mapped reads (%)
Patient	10,216,912	97.0
Mother	13864,179	99.7

Table S8. Specific transcripts found in patient's blood

Position			Expression level (FPKM)	
Start	End	Transcript ID	Patient	Mother
14,216,129	14,228,372	known/full/RBM11/ENST00000400577.4/0	2.0526	0
14,216,129	14,228,372	known/partial/RBM11/ENST00000468643.5/0	0.9330	0
14,273,798	14,291,385	novel/novel_exon_length/ABCC13/-/1	0.5598	0

Table S9. Number of methylated CpG sites in centromeric regions, oscillated region, and other regions

Chromosome	Chr13		Chr14		Chr15		Chr21			Chr22	
Regions	Centromere	Other	Centromere	Other	Centromere	Other	Centromere	Oscillated	Other	Centromere	Other
CpG sites	860	918,195	38	20,173,523	13	86,371	8,062	40,814	38,603	417	59,721
Significant sites	14	1,916	0	1,808	0	1,792	318	928	885	6	1,082
MHS	4	961	0	885	0	927	216	509	465	4	520
PHS	10	955	0	923	0	865	102	419	420	2	562

MHS, maternally hypermethylated CpG sites; PHS, paternally hypermethylated CpG sites

Table S10. Annotation and number of methylated CpG sites in centromeric region of chr21

Number	Simple repeat	LTR	L1	*Alu*	Alpha satellite
CpG sites	710	265	1164	1654	169
Significant sites	34	9	41	57	11
MHS	27	6	26	44	11
PHS	7	3	15	13	0

MHS, maternally hypermethylated CpG sites; PHS, paternally hypermethylated CpG sites; LTR, long terminal repeats; L1, L1 transposon

Table S11. Annotation and number of methylated CpG sites in oscillated and other regions of chr21

	Simple repeat		LTR		L1		*Alu*		Gene		Intergenic unique region	
Number	Oscillated region	Other regions	Oscillated region	Other regions	Oscillated region	Other regions	Oscillated region	Other regions	Oscillated region	Other regions	Oscillated region	Other regions
CpG sites	561	2,539	1,757	770	5,658	2,427	10,505	7,797	14,535	20,650	31,849	29,015
Significant sites	32	53	58	12	148	48	187	143	341	511	896	832
MHS	19	30	23	8	90	29	106	76	196	242	490	435
PHS	13	23	35	4	58	19	81	67	145	269	406	397

MHS, maternally hypermethylated CpG sites; PHS, paternally hypermethylated CpG sites; LTR, long terminal repeats; L1, L1 transposon

Table S12. Primer sequences for qPCR

Gene	Forward primer	Reverse primer
RBM11	TCTTCTGAACCAGCTAACCAAAG	TGGGAAAGGAAGATCTGCC
CXADR	GTGCTCCTGTGCGGAGTAG	ATGGCAGATAGGCAGTTTCC
BTG3	CGCAAGTCCTGTGTACCAGAT	GCCATTCCCTCGATACATTCC
C21orf91	ATTCAGGTGCCACTTTATGTCG	GCCTCTGGACTAGAGATTGTCTC
NRIP1	ATGCAGCAAAGCGGAAGAG	CCTTTAGGCACACTGTCAACC
GABPA	AAGAACGCCTTGGGATACCCT	GTGAGGTCTATATCGGTCATGCT
ATP5PF	ATAAGGAACTTGATCCTATACAGAAACTC	ATACTCTGAACTAGCATCAACAGGTC

Table S13. Expression levels and number of methylated CpG sites in promotors of overexpressed coding genes

	Expression level (FPKM)			Number of CpG sites in promoter	
Gene	Patient	Mother	Ratio	Total	Significant
RBM11	3.843	0.525	7.315	38	0
ABCC13	27.329	10.395	2.629	10	0
HSPA13	49.570	14.639	3.386	0	0
SAMSN1	32.623	12.953	2.518	43	0
AF127936.2	0.549	0.032	17.061	0	0
POLR2CP1	0.794	0	Inf	0	0
AF127936.1	3.561	1.118	3.187	0	0
AF127577.4	2.017	1.141	1.767	0	0
LINC02246	0.392	0	Inf	7	0
AF127577.3	0.354	0	Inf	0	0
NRIP1	29.743	11.717	2.539	10	0
AF127577.2	3.347	1.437	2.328	0	0
AF127577.6	5.974	1.592	3.753	0	0
RAD23BP3	0.729	0.183	3.986	0	0
USP25	92.140	29.539	3.119	187	3
RBPMSLP	4.297	1.727	2.488	0	0
CXADR	0.352	0.059	6.004	95	3
RF00019	0	0.481	0	0	0
BTG3	3.849	1.292	2.980	46	0
C21orf91-OT1	1.942	0.469	4.141	0	0
C21orf91	45.689	15.837	2.885	0	0
AL109761.1	8.878	2.227	3.987	0	0
CHODL-AS1	1.042	0.375	2.780	2	0
AF130417.1	0.782	0.220	3.557	0	0
PPIAP22	201.548	201.548	1	0	0
LINC02573	6.697	2.452	2.731	9	0
LINC01684	0.771	0.663	1.163	14	0
AP000233.1	0	0.359	0	0	0
RPL13AP7	3.180	3.304	0.962	0	0
MIR155HG	3.820	1.632	2.340	76	1
LINC00515	2.040	0.739	2.759	7	0
MRPL39	18.107	7.906	2.290	8	0
ATP5PF	10.396	4.523	2.298	8	0
GABPA	31.115	14.533	2.141	89	0
LLPHP2	1.180	0.780	1.512	0	0

Inf, infinite
